# Beyond the Distributed Practice Effect: Is Distributed Learning Also Effective for Learning With Non-repeated Text Materials?

**DOI:** 10.3389/fpsyg.2021.685245

**Published:** 2021-10-15

**Authors:** Carla Elisabeth Greving, Tobias Richter

**Affiliations:** Department of Psychology IV, University of Würzburg, Würzburg, Germany

**Keywords:** distributed practice, learning from text, retention interval, spacing effect, reading

## Abstract

Distributed learning is often recommended as a general learning strategy, but previous research has established its benefits mainly for learning with repeated materials. In two experiments, we investigated distributed learning with complementary text materials. 77 (Experiment 1) and 130 (Experiment 2) seventh graders read two texts, massed vs. distributed, by 1 week (Experiment 1) or 15 min (Experiment 2). Learning outcomes were measured immediately and 1 week later and metacognitive judgments of learning were assessed. In Experiment 1, distributed learning was perceived as more difficult than massed learning. In both experiments, massed learning led to better outcomes immediately after learning but learning outcomes were lower after 1 week. No such decrease occurred for distributed learning, yielding similar outcomes for massed and distributed learning after 1 week. In sum, no benefits of distributed learning vs. massed learning were found, but distributed learning might lower the decrease in learning outcomes over time.

## Introduction

Learning from texts is crucial for knowledge acquisition in school and in higher education. However, rereading texts is time-consuming and does not necessarily lead to successful learning (Dunlosky et al., 2013). Thus, exploring strategies that can improve learning from text is an important research focus. One central question is whether this goal can be accomplished better by making the comprehension process easier or by making it more difficult, which might engage the reader in deeper processing of the text (McNamara et al., 1996). The latter strategy is consistent with the desirable difficulties approach. Desirable difficulties are properties of learning procedures that make the learning process subjectively difficult, which may hamper learning in the short run but foster better long-term retention (Bjork, 1994; Bjork and Bjork, 2011; Lipowsky et al., 2015). Desirable difficulties might be involved in different learning procedures such as retrieval practice and distributed practice. Regarding the underlying cognitive mechanisms, several theories assume that the retrieval and activation of prior knowledge during learning is important for making a learning difficulty desirable (Delaney et al., 2010; Bjork and Bjork, 2011; Toppino and Gerbier, 2014).

In learning from text, a desirable difficulty can be introduced by distributing the rereading of text materials, thus, reading the same material for the second time, over time. Distributed rereading has been shown to enhance learning outcomes in the long run (in free recall *d* = 0.56, Rawson, 2012; in free recall *d* = 0.73, in text comprehension performance *d* = 0.53 Rawson and Kintsch, 2005). However, apart from self-regulated learning for an exam or test, the application of distributed rereading in learning situations is limited because it only focuses on repetition. In classroom learning, reviewing materials is uncommon (Dempster, 1989). For example, when reading a textbook in school, texts are rarely read repeatedly but instead are followed by reading new texts (e.g., an advanced chapter in a textbook) that are related to texts read earlier (e.g., an introductory chapter in a textbook). The extent that the distribution of learning time functions as a desirable difficulty when reading two or more texts that are complementary to each other remains an open question.

In the present research, we investigated whether distributed reading of two complementary texts (similar to subsequent chapters in a textbook) makes learning from these texts more difficult for school students but simultaneously improves long-term retention. We assumed that the information given in the first text must be retrieved when reading the second text to establish coherence, which is more difficult when the texts are distributed over time. This retrieval difficulty should lead to better text recall, especially in the long run. In the following sections, we discuss the relevant processes underlying learning with texts. We discuss the effects of prior knowledge and how information is (re)activated when reading. Based on a discussion of distributed practice, we propose the idea of distribution by time in learning with complementary texts.

### Improving Learning From Text

Learning from text is based on comprehending the text, which involves the construction of a situation model (also termed mental model) of the text content. The situation model goes beyond a representation of the text itself (surface representation) or the information explicitly given in a text (propositional textbase, Kintsch, 1994). Deep understanding of texts and long-term learning might benefit especially from the active retrieval of information from long-term memory, which can be interpreted according to the principle of generative learning (Wittrock, 2010) or learning as active information processing (Mayer, 1996). In their landscape model of reading, van den Broek et al. (1996) termed this process as coherence-based retrieval. Contrary to the process of cohort activation, which is based on a passive spread-of-activation mechanism, coherence-based retrieval is a strategic, slow, and effortful process that aims at establishing coherence in accordance with readers’ goals and standards of coherence.

Previous research has examined numerous possibilities to improve learning from text by increasing the difficulty of text processing and, arguably, by promoting active, coherence-based retrieval during comprehension. Several studies by McNamara and colleagues looked at the effects of lowered text cohesion on learning. These studies found that better (i.e., more cohesive) texts improve the comprehension of students with low prior knowledge, whereas the opposite was found for high-knowledge students (reverse coherence effect, McNamara and Kintsch, 1996; McNamara et al., 1996). Low-cohesion texts seem to foster the active retrieval of prior knowledge and knowledge-based inferences of high-knowledge students, which might account for their higher comprehension outcomes (McNamara, 2001). However, this effect also seems to depend on reading skill, as skilled readers also have been shown to profit from high-cohesion texts (Ozuru et al., 2009). Therefore, especially less skilled readers need low cohesion texts to activate their prior knowledge.

Other text features have been varied to assess the extent that they stimulate active processing of information such as local and global coherence (Boscolo and Mason, 2003), syntactic structure of sentences (Feng et al., 2013), presentation format, text organization and example context (McCrudden et al., 2004), sentence order and letter deletion (McDaniel et al., 2002), and verb cohesion and syntactic simplicity (Mills et al., 2015), but these studies have yielded mixed results of increased processing difficulty, ranging from positive effects to negative effects on learning. Apart from obvious differences in the manipulation of text difficulty, one condition that might have contributed to the inconsistent results is that learning outcomes were measured immediately after reading in these studies. But desirable difficulties might play out their advantages in particular at longer intervals between learning and assessment of learning outcomes (Rawson and Kintsch, 2005; Pashler et al., 2007; Rawson, 2012; but see also Dunlosky et al., 2013).

### Distribution of Text Reading as Special Case of Distributed Practice

Distributing text reading over time might be another largely unexplored possibility to promote active processing and especially to retrieve information when learning from texts. The effects of temporal spacing of materials have been studied extensively with regard to distributed practice in which repetitions of the same materials (or repetitive practice of similar materials) are distributed into several (shorter) learning sessions rather than one (longer) learning session (massed practice). For this type of learning, spacing usually has positive effects, especially for long-term retention, a phenomenon called the spacing effect (Cepeda et al., 2006). Longer interstudy intervals (i.e., the time between repetitions) are usually better for longer retention intervals, a phenomenon also known as the lag effect (Cepeda et al., 2006, 2008; Rohrer, 2015). In accordance with Cepeda et al. (2006) and Küpper-Tetzel (2014), we will use the term distributed practice to refer to both effects, thus, the spacing and the lag effect.

Positive effects of distributed practice have mainly been shown for simpler materials such as word pairs or learning the vocabulary of a foreign language (Cepeda et al., 2006, 2008, 2009), but some studies have also established distributed practice effects for more complex materials such as science concepts (Vlach and Sandhofer, 2012; Vlach, 2014) and expository texts (Rawson and Kintsch, 2005; Verkoeijen et al., 2008; Rawson, 2012; but see Greving and Richter, 2019, who did not find a benefit in seventh graders). Thus, distributed practice seems to be beneficial for learning with a broad range of materials.

Overall, the benefits of distributed practice are a robust empirical phenomenon (Cepeda et al., 2006; Carpenter et al., 2012). Donovan and Radosevich (1999) reported in their meta-analysis an overall mean weighted effect size (Cohen’s *d*) of 0.46 (95% CI [0.42, 0.50]). In their review of learning techniques, Dunlosky et al. (2013) evaluated distributed practice as having high utility for learning. Despite these findings, students seem to underrate the effectiveness of distributed practice (see Son and Simon, 2012 for a review). For example, when learners were asked immediately after learning to estimate the proportion of items they would correctly recall in a posttest, their estimates were higher for items learned in a massed fashion compared to items learned in a distributed fashion (Kornell, 2009). One possible explanation for the negative effects of distributed practice on the meta-cognitive judgment of predicted learning success might be a lower experienced fluency during distributed practice (Alter and Oppenheimer, 2009; Bjork et al., 2013), which would be in line with the interpretation that distributed practice could induce a desirable difficulty.

Schwartz et al. (2011) defined distributed practice as “learning that is spread out across relatively long periods of time rather than massed all at once” (p. 10). This definition suggests that the benefits of spacing might not be restricted to learning materials that are repeated explicitly but also extend to learning with related but not repeated materials. Nevertheless, currently, the evidence for beneficial effects of distributing non-repeated learning materials over time is scarce. To prevent misunderstandings, we will use the term distributed learning to refer to the distribution of learning materials that are not repeated and the term distributed practice for learning that involves the distribution of repeated learning materials. Please note that the term distributed learning might also be understood as a superordinate term that subsumes both forms of learning with materials that are distributed overtime but that we use it in a more specific way here to designate distributed learning with non-repeated materials.

Especially, we are interested in distributed learning with multiple, complementary texts. At this point, it is useful to define the notion of text. We use a broad definition of text here according to which a “text represents the inscription of ideas in linguistic form” (Alexander and Jetton, 2003, p. 201). Thus, texts are made of written or spoken words, possibly accompanied by other modes of representation such as graphs, pictures, or animated pictures. We speak of a “text” when it can stand on its own, that is when the average reader can, in principle, establish a globally coherent, meaningful representation of the text content by reading the text (or listening to it) and drawing on their prior knowledge. Thus, textbook chapters usually qualify as texts in the sense of this definition, whereas, for example, paragraphs within a chapter would not be texts because they do not make sense when read on their own. That said, the example of textbook chapters shows that reading one text can be particularly helpful to understand another one. School learning often involves the reading of multiple texts that cover different aspects of the same topic such as subsequent chapters in a textbook. Those texts can be framed as complementary texts. Complementary texts are multiple texts that are “convergent and require adding pieces of information together” (Primor and Katzir, 2018, p. 4; see also Richter et al., 2020). As Carpenter et al. (2012) noted, textbooks typically do not provide distributed repetition of concepts. Content provided earlier in the textbook often serves as background knowledge that is helpful for understanding later chapters. For example, the first lesson in a science class that covers the complex topic of cell biology might require students to view the plant cell under the microscope and then consult their textbook to read information about the different components of the cell (e.g., the functions of different organelles). In the next lesson, students might read the subsequent textbook chapter about the bacterial cell. In this chapter, they would learn about the structure of the bacterial cell and which organelles can be found in the bacterial cell. However, in this chapter, the functions of the organelles will not be explained again. Thus, the students must retrieve this information from memory to fully understand the chapter. The time between lessons could also vary. The second lesson might follow immediately after the first one (massed learning) or after some time has elapsed between the two lessons (distributed learning). This scenario leads to the question of whether the well-established benefits of the distribution of learning also occur for reading complementary but non-repeated text materials in the school learning environment.

An indication that distributed practice effects may occur even without repetition was provided by Braun and Rubin (1998, Experiment 3). In this experiment, the participants learned word lists with massed and distributed presentation of word pairs. The two words that formed a pair began with the same three letters. For example, if the first word was BURden, the second was BURlap (example from Delaney et al., 2010). Although the second word was not an exact repetition of the first word (i.e., only a partial overlap), a spacing effect occurred for the first and the second word. A study by Vlach and Sandhofer (2012) is another example of distributed learning without explicit repetition. Notably, these authors used more complex materials in a real-world educational setting. They investigated whether distributed practice can aid the generalization of science concepts in children. Elementary school students were taught the concept of food chains. The children received four lessons in massed (all sessions in immediate succession on 1 day), clumped (two sessions on 1 day, two sessions on the next day) or spaced (one session per day for 4 days) schedules. In each of the lessons, the food chain was illustrated within a different biome. Thus, the materials were not repeated exactly as in typical distributed-practice studies, but the concept of food chains was repeatedly embedded in different contexts. 1 week after the final learning session, the ability to make simple and complex generalizations of the concept to a new biome was assessed with two tasks. Children with a distributed learning schedule outperformed children with a massed learning schedule in both tasks (*d* = 0.89, respectively, *d* = 1.91, calculated from η^2^). Another study of distributed learning with non-repeated materials was conducted by Smith and Rothkopf (1984). In an 8-h statistics course, parts of the course (four videotaped sessions) were presented in a distributed fashion with an interstudy interval of 1 day or massed within 1 day with only short breaks between the sessions. After 5 days, participants who received the distributed sessions outperformed participants who received the sessions in a massed fashion in free and cued recall with an increase of 13% and 14%, respectively. In a more recent study, Randler et al. (2008) investigated blocked vs. traditional teaching in biology class. The traditional condition received four lessons of 45 min each in a weekly schedule, while in the blocked condition, the students received all lessons within one morning. Immediately after the last lesson, students in the traditional condition outperformed students in the blocked condition, whereas no difference was found 7 weeks after the last lesson. In sum, these studies provide evidence of distributed learning effects with non-repeated materials. However, to our knowledge, no research exists that has examined distributed learning effects in learning with multiple, complementary texts.

## Rationale of the Present Experiments

**FIGURE 1:**
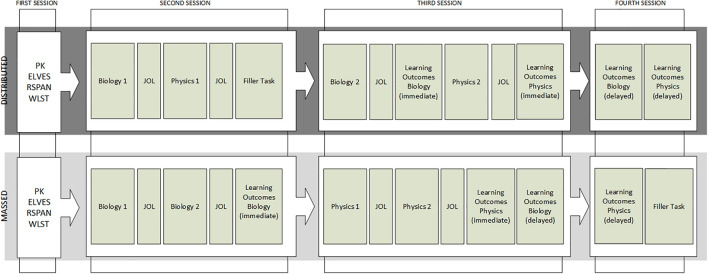
Procedure of Experiment 1. An illustration of the procedure of Experiment 1. Order of topics was counterbalanced between students (only one example is shown here). PK = Assessment of prior knowledge; ELVES = Assessment of reading ability; RSPAN = Assessment of working memory; WLST = Assessment of reading strategy knowledge; Biology 1 = first text biology; JOL = assessment of meta-cognitive judgments of the learning process; Physics 1 = first text Physics; Biology 2 = second text biology; Physics 2 = second text physics; Learning outcome physics = assessment of learning outcome for physics; and Learning outcome biology = assessment of learning outcome for biology. Learning outcomes were measured immediate after learning (immediate) and 1 week delayed (delayed).

We conducted two experiments to test the assumption that the temporal distribution of complementary multiple texts leads to better learning. Both experiments were conducted with students in Grade 7 (12–13 years old) in the school classroom. The experimental materials covered two different domains to gain tentative information about the generalizability of results. In Experiment 1, participants received the texts from both domains (within-subjects, Figure 1). In Experiment 2, each participant received only the texts from one domain, that is, topic was varied between-subjects.

Two pairs of expository texts from the natural sciences (biology and physics) were developed to match the typical contents and difficulty of texts that seventh graders read in their regular classes. The texts were coherent with each other in the sense that the second text built on concepts from the first text, resembling subsequent textbook chapters. Learners were randomly assigned to one of two learning conditions. They read the two texts per domain in a massed fashion or in a distributed fashion with a learning interval of 1 week (Experiment 1) or 15 min (Experiment 2) between the two texts. Immediately after learning, students judged four aspects of the learning process. They indicated the perceived difficulty of the reading task and predicted their learning success. Furthermore, they rated the perceived similarity between the texts and perceived learning coherence. Learning outcomes were assessed approximately 5 min after learning (immediate) and 1 week later (delayed).

The following hypotheses were derived from the theoretical considerations laid out in the previous sections:

Hypothesis 1 (main hypothesis): We expected the potential learning benefits of distributed learning for learning from both texts to depend on time of test. Immediately after learning (that is immediately after reading the second text), we expected no benefits of distributed over massed reading (Hypothesis 1a), whereas a learning benefit of distributed reading should emerge at a longer time interval of 1 week after learning (Hypothesis 1b).

Hypothesis 2: We expected domain-specific prior knowledge to be a positive predictor of learning outcomes. Text comprehension and learning from text are based on integrating new information with existing knowledge (Kintsch, 1988). In line with this general notion, numerous empirical studies found domain-specific prior knowledge to be a strong predictor of text comprehension and learning from text (e.g., Schneider et al., 1989; Ozuru et al., 2009).

Hypothesis 3: We expected that distributed learning – as desirable difficulty – should lead to overall higher perceived difficulty (Hypothesis 3a) and lower expected learning success (Hypothesis 3b) compared to massed reading.

Hypothesis 4: Finally, we expected the perceived similarity (Hypothesis 4a), that is how similar participants judged the texts read at the two learning occasions, and learning coherence (Hypothesis 4b), that is as how strong they judged the relationships of the two text, to be lower in the distributed than in the massed condition. This hypothesis can be derived from the assumption that the passive retrieval of information is more difficult and therefore less likely to be successful in distributed learning.

In addition to testing these hypotheses, three learner characteristics, reading ability, working memory capacity, and (in Experiment 1) reading strategy knowledge were examined to control for pre-existing differences in these variables.

## Experiment 1

Experiment 1 examined distributed learning with a 1-week interval between reading the first and second text in the distributed condition, as opposed to no interval in the massed condition. The time interval was chosen because school lessons often follow a weekly schedule, which makes a 1-week learning interval an ecologically valid interval with which to start. Moreover, learning intervals of 1 week have been used in previous studies on distributed rereading (e.g., Rawson and Kintsch, 2005).

### Method

#### Design

Experiment 1 was based on a 2 × 2 × 2 design with the independent variables learning condition (massed vs. distributed learning), retention interval (immediate vs. 1-week delay), and domain (biology vs. physics). Learning condition was varied between participants, retention interval and domain were varied within participants. Participants were randomly assigned to one of the two learning conditions within classes, thus, all experimental conditions were realized in each class. The assignment of the two different comprehension tests to one of the two retention intervals was counterbalanced between participants.

#### Participants

Ninety-seven seventh graders (52 boys, 45 girls) with a mean age of 12.32 years (*SD* = 0.47, age was not reported for nine students) from four classes of a German comprehensive school participated in the experiment. Parental permission was obtained for all participating students. Students without permission did not participate in the experiment. For those students, no data was recorded due to data protection regulations. Therefore, we have no information how many students did not receive their parents’ permission to participate in the study. Students were randomly assigned to either the massed learning condition (*n* = 50) or distributed learning condition (*n* = 47). Students received sweets after each of the sessions and a magic cube puzzle after the last session as a reward for participation. Fourteen students missed one of the two learning sessions and were excluded from all analyses. Four students missed the first session in which the domain-specific prior knowledge test was assessed. Their data was excluded from analysis. The data of one student was excluded because of technical problems. In the end, 77 participants (massed learning condition: 38; distributed condition: 39) remained in the sample.

#### Power Analysis

This study was the first to investigate distributed reading with complementary text materials, which made it impossible to form expectations about effect sizes based on previous studies. The experiment was conducted in the classroom with a heterogeneous sample, which is likely to limit possible effects. Therefore, we based power calculations on the assumption of a small population effect (*d* = 0.3 or OR = 1.72, respectively), following Cohen’s conventions for effect sizes (Cohen, 1988). The power (1-β) for finding an interaction effect between learning condition and retention interval of this size, determined by simulation with the R package simr (Green and MacLeod, 2016), was high (1.000, 95% CI [0.996, 1.000]) given the assumed Type-I error probability (α) of 0.05.

#### Text Materials

Two experimental texts were developed for each of the two domains. For the biology domain, the first text explained the plant cell and its components, and the second text explained the bacterial cell. For the physics domain, the first text explained the law of conversation of energy, and the second text explained the first law of thermodynamics. The length of the texts ranged from 504 to 633 words and the Flesch reading ease (German formula, Amstad, 1978) ranged from 46 to 60. The biology texts contained images illustrating the structure of the respective cell; this image was presented during the whole text. We added this image to enhance comprehension of the cell structure and to enhance the ecological validity of the text, as expository texts in biology usually contain images. The first physics text contained an illustrative image of an experiment by James Prescott Joules, which was explained in the text. See Supplementary Appendix A for translations of the texts used in the experiment.

The texts were constructed as self-containing texts, comparable to two chapters in the same textbook. The texts were related to each other, but Text 2 was still comprehendible without reading the Text 1, provided that the relevant prior knowledge was available. Nevertheless, in Text 1, some basic information was provided, which was relevant for understanding Text 2 but not repeated in this text. For example, in the set of biology texts, the function of the ribosomes were explained in Text 1, but not in Text 2, even if in Text 2 it is mentioned that bacterial cell have ribosomes as well. In the set of the physics text, is was explained in Text 1 what the term closed system means, but in Text 1, the definition was not repeated, although the term is needed to understand the concept of internal energy. The topic of both the biology and the physics texts were chosen after consultation with teachers. The criteria were that the topics should be optional parts of the school curriculum that are not taught regularly in school. We also made sure that the topics were indeed not taught in the participating classes. Therefore, we considered the prior knowledge to be low enough to (1) be able to acquire new knowledge by reading the texts and (2) make Text 1 relevant for full comprehension of Text 2.

#### Assessment of Learning Outcomes (Text Comprehension)

For each domain, two comprehension test forms (A and B) were constructed to assess learning outcomes. Each test form contained eight short-answer questions and seven multiple-choice questions (one correct response, three distractors). The two different types of questions were used because of their different requirements regarding memory processes (cued recall and recognition) and the accompanying differences in item difficulty. Each student received each test form, counterbalanced at either the immediate or the delayed test. The different test forms were constructed to ensure that different questions are posed at the two times of tests. The questions were constructed in pairs (except two questions in biology), thus, the questions differed in their wording (and/or type) but referred to the same information.

For example, one short-answer question was, A bacteria cell does not have a cell nucleus. But where can you find the genome of the bacteria cell?, and one multiple-choice questions was, To which kind of organism does the bacteria cell belong?, with the response options (a) Prokaryotes, (b) Eukaryotes, (c) Plasmid, and (d) Organelle. The order of questions was randomized. All answers to the short-answer questions were scored as either incorrect (0) or correct (1) by two independent raters who were blind to the experimental conditions (Cohen’s κ = 0.91). In the few instances of disagreement (0.5%), the score provided by one of the two raters (determined randomly) was used.

The questions could be answered based on information from both texts (12%), from Text 1 (45%), or from Text 2 (43%). The questions referring to Text 1 and 2 asked for information explicitly given in the respective text. The questions referring to information from both texts made a form of intertextual inference necessary, such as comparing plant and bacterial cell.

In a pilot study, 82 students from 3 classes of a comprehensive school read the two texts of one of the two domains (randomly assigned) in massed fashion. Afterward, they answered questions (33 in biology, 28 in physics) and solved a cloze with 12 gaps. Following the feedback of the teacher of the classes as well as the item difficulties and conceptional reasons, we decided to remove the cloze from the test and revised the questions intensely. 11 questions were removed (7 biology questions, 2 physics questions). One biology question was divided into two parallel questions and 7 multiple-choice questions (3 biology questions, 4 physics questions) were additionally created paralleling tested short-answer questions. Furthermore, all questions were revised in wording, adding some background to the questions to increase the retrievability. For example, one question was Why is the golgi apparatus called post office?. In revision, we added the following background information: Plant cells have an organelle, which is called golgi apparatus. The golgi apparatus is also called post office as introduction of the question and added the suffix Justify your answer.

Nevertheless, the items were still difficult, with a mean item difficulty of 0.20 (*SD* = 0.16) in the short-answer questions and a mean item difficulty of 0.41 (*SD* = 0.15) for multiple-choice questions. Cronbach’s α for the different learning outcomes tests ranged from 0.71 and 0.59 (physics form A and B) and 0.72 and 0.62 (biology form A and B).

#### Assessment of Domain-Specific Prior Knowledge

For the biology assessment, the participants answered two short-answer questions about basic terms that appeared in the text. Additionally, they received images of the cell structures of the bacterial and plant cell and were required to label the components of the cells. For the physics assessment, the participants answered five short-answer questions about basic terms that appeared in the text. The different amount of questions was chosen because we assumed that the labeling questions would take longer and produce more variance than the short-answer questions. The order of the domains and questions within domains were randomized. One third of the responses were scored by two independent raters (Cohen’s κ = 0.75). The internal consistency was low (biology: Cronbach’s α = 0.57 95% CI [0.43,0.65]; physics: Cronbach’s α = 0.38, 95% CI [0.17,0.54]). However, as the questions were developed to cover the curriculum-orientated knowledge within the two domains, the questions differ relative broad in topic [e.g., one question about the (plant) cell, one question about the genetic makeup]. For a curriculum-based knowledge test like this, internal consistency might not be the most informative way to estimate reliability (Schmitt, 1996). Moreover, prior knowledge was generally low in the present sample, which restricts the item variance and, hence, the inter-item correlations that the internal consistency is based on. Considering these circumstances, we decided to proceed with the prior knowledge measure despite the low internal consistencies.

#### Assessment of Further Learner Characteristics

To control for pre-experimental differences between the experimental groups, we assessed several learner characteristics. In a teacher questionnaire, we asked the teachers to provide the students’ grades in biology and physics (ranging from 1 = “very good” to 6 = “unsatisfactory”) along with other learner characteristics such as age. Knowledge about reading strategies was assessed with the Würzburger Lesestrategie Test (WLST; Würzburg Reading Strategy Test; Schlagmüller and Schneider, 2007; split-half reliability: *r* = 0.90, estimated in a sample of 4,490 students in Grades 7–11). Reading ability was assessed with the subtest sentence verification of the German-speaking test of reading abilities ELVES (Richter and van Holt, 2005; Cronbach’s α = 0.83 assessed in the current sample) and working memory with a computerized version of a Reading Span Task (RSPAN; Oberauer et al., 2000; Cronbach’s α = 0.86, assessed in the current sample). These learner characteristics were included only to control for differences between learning conditions.

#### Metacognitive Judgments of the Learning Process

After each text, participants judged several aspects of the reading process on 5-point Likert scales. They made a prediction of their learning success (What do you think, how well will you remember the content of the text you just read?; response options ranging from 1: very bad to 5: very good) and rated the perceived reading difficulty (How difficult was it for you to read the text?; response options ranging from 1: very difficult to 5: not difficult at all). After reading the second text, they rated the perceived similarity of the texts (three items, e.g., The structure of the two texts was very similar, Cronbach’s α = 0.97, response options ranging from 1: not true to 5: true) and the perceived learning coherence (Reading the second text helped me to understand what the first text was about, response options ranging from 1: not true to 5: true).

#### Procedure

All materials were presented on notebook computers (16.6” screen) and with the software Inquisit 3, 2011 (*Inquisit 3 [Computer Software]* (Version 3.0.6.0)). The experiment was conducted in the classroom and consisted of four sessions (Figure 1). In the first session, students received examples for the different question types and an example of the reading task. Afterward, the participants completed the four tasks related to learner characteristics: the assessment of domain specific prior knowledge, the WLST, the ELVES, and the RSPAN tests. In some classes, the RSPAN or the ELVES or both could not be conducted during the pretest session. In this case, the tests were conducted at the end of the final session.

The procedure at the remaining three sessions varied depending on the learning condition. In the second session, the participants were randomly assigned to the learning conditions. In the second and third session, the participants either read two texts in one domain (massed condition) or one text each of the two domains (distributed condition). Learning outcomes for each domain were assessed immediately after reading the second text in the domain and 1 week later.

All sessions started with a general instruction read aloud by the student research assistant who conducted the experiment. The instructions used in Experiment 1 are displayed in Supplementary Appendix C. In the pretest, the instruction of each task was read aloud. For the following sessions all instructions were presented on screen. Participants were informed that they would read multiple texts. However, they did not know when they would read the texts and when the respective tests would take place. Thus, the participants were not aware about the assignment to different reading conditions. During the sessions, two instructors were present to help with technical problems and to ensure that all participants were working quietly. The participants read the experimental texts with the moving-window-method in a self-paced fashion. While reading, all sentences except the one that participants were currently reading were blurred. Thus, they could only read one sentence at a time. Participants were able to advance to the next sentences by pressing a key and to return to the previous sentences for rereading by pressing another key.

In two classes, the sessions could not be conducted as scheduled, which resulted in fewer days between Session 2 and Session 3 or between Session 3 and Session 4. Consequently, 10 participants in the distributed condition read the texts with a learning interval of 3 days instead of 1 week. For 10 other participants, the retention interval for the delayed test for at least one of the domains was only 3 days, and for 21 participants the delay was 6 days instead of 1 week. To examine the impact of these deviations from the experiment schedule, we ran all analyses regarding learning outcomes with and without the data of these participants. The effects remained unchanged. Therefore, we report the results for the full data set.

#### Data Analysis

We used generalized linear mixed effect models (GLMMs) for analyzing the effects of the independent variables on learning outcomes. The GLMM analyses were performed with the R packages lme4 (Bates et al., 2015), lmerTest (Kuznetsova et al., 2017), and lsmeans (Lenth, 2016). GLMM was used because of the multilevel structure of learning outcomes. A multilevel structure is typical for experiments where a sample of participants work on a sample of experimental items (Baayen et al., 2008). This was the case in our experiments, in which the participants answered 60 questions. A multilevel structure is also characteristic for classroom studies where students come from different classes. Such multiple levels create dependencies in a data set which are basically ignored by one-level analysis methods such as ANOVA or traditional multiple regression analysis. Consequently, using these methods can be misleading, among other things by underestimating standard errors and causing false-positive significance tests (for a discussion for continuous outcome variables see Richter, 2006; Baayen et al., 2008; for categorial outcome variables see Dixon, 2008; Jaeger, 2008).

We included class, participant and item as random effects (random intercepts) when the intra-class correlation of a dependent variable (a measure to quantify interdependencies in the data) exceeded 0.05. Thus, models with random intercepts but with no random slopes were estimated. Such models bear the risk of inflating Type-I error (Barr et al., 2013). However, including more random effect variance components also decreases power and easily overtaxes the information available in the data, leading to misspecified models that cannot be estimated (Matuschek et al., 2017). The fixed-effect structure of our models was already quite complex, making it impossible to estimate several random slopes and their covariances. Therefore, we estimated models with random intercepts only. For all models, the distribution of residuals was inspected visually for normality. For the interpretation of GLMM results, the predicted probabilities (back-transformed from the log odds) are reported. Type I error probability was set at 0.05 for all hypothesis tests. Directed hypotheses were tested with one-tailed tests.

### Results

#### Differences Between Experimental Groups

There were no group differences between the two learning conditions with regard to working memory capacity, *t*(66.06) = -1.48, *p* = 0.144, knowledge about reading strategies, *t*(54.28) = 0.13, *p* = 0.962, or the teacher-reported grades in biology, *W* = 832.5, *p* = 0.122, and physics, *W* = 820.5, *p* = 0.371. However, the groups differed in their reading ability. The participants in the massed condition outperformed the participants in the distributed condition, *t*(65.48) = -2.35, *p* = 0.022. Therefore, we controlled for reading ability in all analyses. Table 1 provides the means and standard deviations of all dependent variables and learner characteristics observed in the two experimental groups. Correlations of all measured variables (including correlations within the learning conditions) are provided in Supplementary Appendix Table B1.

**TABLE 1:** Means and standard deviations of dependent variables and learner characteristics in Experiment 1.

	Biology				Physics			
	Massed		Distributed		Massed		Distributed	
Variable	*M*	*SD*	*M*	*SD*	*M*	*SD*	*M*	*SD*
Dependent variables								
Learning outcome (immediate)	5.95	3.19	4.37	2.47	4.76	2.92	2.93	2.93
Learning outcome (delayed)	4.59	2.78	4.07	2.02	3.63	2.05	3.14	1.73
Perceived difficulty	3.38	0.80	3.35	0.72	3.02	0.82	3.01	0.69
Self-predicted success	2.94	0.70	2.73	0.73	2.72	0.68	2.43	0.59
Perceived similarity	3.34	0.67	3.02	0.65	3.35	0.54	2.95	0.75
Perceived learning coherence	2.90	1.16	2.26	0.95	2.92	0.92	2.36	1.10
Domain-specific learner characteristics								
Domain-specific prior knowledge	2.78	2.42	2.93	2.32	3.56	1.92	5.19	2.26
Grades	2.56	0.71	2.48	0.89	2.28	0.70	2.29	0.85

**Further learner characteristics**	**Massed**				**Distributed**			
	***M***		***SD***		***M***		***SD***	

Reading ability	17.16		5.53		14.09		4.28	
Working memory capacity	0.61		0.13		0.54		0.18	
Reading strategy knowledge	54.42		9.83		55.11		11.57	

*All domain-specific variables are provided separately for the domains. Domain was varied within-subjects.*

#### Learning Outcomes

To test Hypotheses 1 and 2, we estimated a generalized mixed model with learning condition (contrast-coded: distributed = 1, massed = -1) and retention interval (contrast-coded: immediate = -1, delayed = 1) with their interaction and domain-specific prior knowledge (*z*-transformed) as predictors. Text comprehension performance served as the dependent variable. In addition, we included domain2 (contrast-coded: physics = 1, biology = -1) and reading ability as predictors for control purposes. Items and participants were included as random effects (random intercepts). All estimates are provided in Table 2.

**TABLE 2:** Parameter estimates and significance tests for the generalized mixed model for learning outcomes in experiment 1.

Fixed effects		
**Predictors**	**Estimate**	***SE***
(Intercept)	−1.41***	0.18
Learning condition	−0.11	0.08
Retention interval	−0.12**	0.04
Prior knowledge (*z*-standardized)	0.15*	0.06
Domain	−0.32*	0.16
Reading ability (*z*-standardized)	0.23**	0.08
Learning condition x Retention interval	0.08^+^	0.04

**Random effects**		

σ^2^	3.29	
τ_00_	0.32 _*ID*_	
	1.43 _*Item*_	

**Goodness of fit**		

Deviance	4157.23	

*Learning condition (contrast-coded: distributed = 1, massed = -1). Retention interval (contrast-coded: immediate = -1, delayed = 1). Domain (contrast-coded: biology = -1, physics = 1). Prior knowledge and reading ability were included *z*-standardized. Directional hypotheses were tested one-tailed.*

*p < 0.05 (two-tailed). **p < 0.01 (two-tailed). ***p < 0.001 (two-tailed).

*^+^*p* < 0.05 (one-tailed).*

**FIGURE 2:**
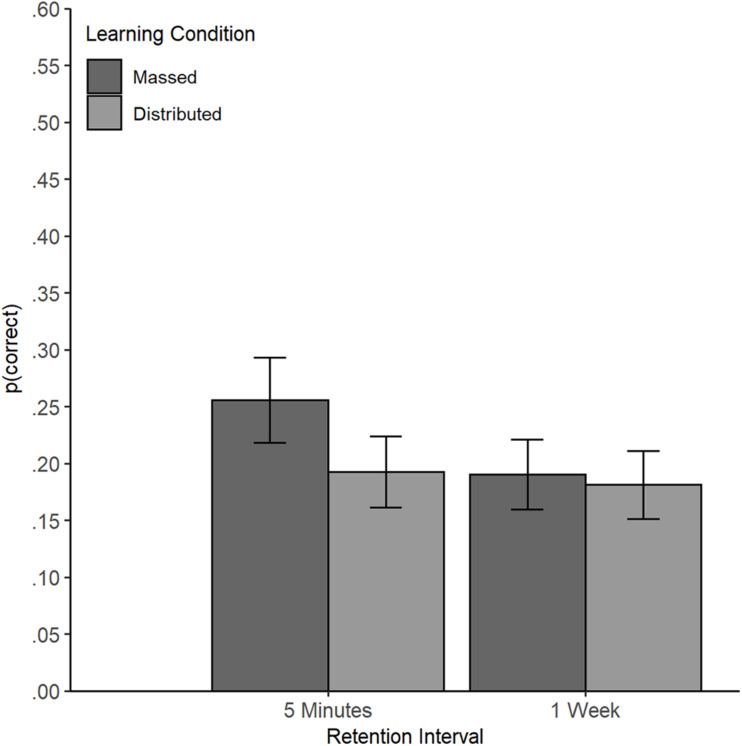
Interaction of Learning Condition and Retention Interval in Experiment 1. Learning outcomes (text comprehension) by learning condition at the short and long retention interval in Experiment 1 (estimated based on Model 1, back-transformed probability of a correct answer). Error bars represent standard errors (±1 SE).

No main effect of the learning condition was found (β = -0.11, *SE* = 0.08, *z* = -1.36, *p* = 0.175). No overall difference in performance was found between the massed condition (probability = 0.22, *SE* = 0.03) and the distributed condition (probability = 0.19, *SE* = 0.03), *OR* = 1.24 (95% CI [0.91, 1.70]). A main effect of retention interval emerged (β = -0.12, *SE* = 0.04, *z* = -2.86, *p* = 0.004) with better comprehension performance in the test immediately after reading the text (probability = 0.22, *SE* = 0.03) compared to the test after 1 week (probability = 0.19, *SE* = 0.03), *OR* = 0.81 (95% CI [0.68, 0.95]). However, this main effect was qualified by an interaction of retention interval and learning condition (β = 0.08, *SE* = 0.04, *z* = 1.92, *p* = 0.027, one-tailed, Figure 2).

Planned comparisons revealed that the learning outcome of participants in the massed condition decreased from the immediate to the delayed test (immediate test: probability = 0.26, *SE* = 0.04; delayed test: probability = 0.19, *SE* = 0.03), *z* = 3.23, *p* = 0.001, *OR* = 0.68 (95% CI [0.55, 0.85]). In contrast, the learning outcomes of participants in the distributed condition remained stable (immediate test: probability = 0.19, *SE* = 0.03; delayed test: probability = 0.18, *SE* = 0.03), *z* = 0.67, *p* = 0.504, *OR* = 0.93 (95% CI [0.74, 1.16]). At the shorter retention interval, the participants in the massed condition outperformed participants in the distributed condition (*z* = -2.06, *p* = 0.040), *OR* = 1.45 (95% CI [1.02, 2.05]). However, at the longer retention interval, no difference between the two learning conditions emerged (*z* = -0.35, *p* = 0.728), *OR* = 1.07 (95% CI [0.75, 1.52]).

Additionally, we found the predicted main effect of prior knowledge (β = 0.15, *SE* = 0.06, *z* = 2.52, *p* = 0.012), indicating that text comprehension performance was positively associated with prior knowledge. A difference of one standard deviation in prior knowledge corresponded to an odds ratio of 1.16 (95% CI [1.03, 1.31]).

In sum, we did not find the benefit of distributed reading predicted by Hypothesis 1. On the contrary, at the immediate test an advantage of the massed condition was found. This advantage disappeared at the delayed test, but it did not turn into an advantage for the distributed condition. In the distributed condition, we found no decrease between the short and the long retention interval. Hypothesis 2, stating that prior knowledge would benefit learning from the text materials was supported by the data.

#### Metacognitive Judgments of the Learning Process

We estimated two multivariate linear regression models with the metacognitive judgments as dependent variables and learning condition (contrast-coded: distributed = 1, massed = -1), text (contrast-coded: first text = -1, second text = 1) and their interaction plus the domain (contrast-coded: biology = -1, physics = 1) as predictors. We used the R package car for the hypothesis tests (Fox and Weisberg, 2019). The variables self-predicted success and perceived reading difficulty were recoded so that higher values correspondent to higher difficulty and lower predicted success, corresponding to Hypothesis 3. Means were estimated from the corresponding univariate linear regression models using the R package lsmeans (Lenth, 2016).

##### Meta-cognitive judgments of predicted success and reading difficulty

**FIGURE 3:**
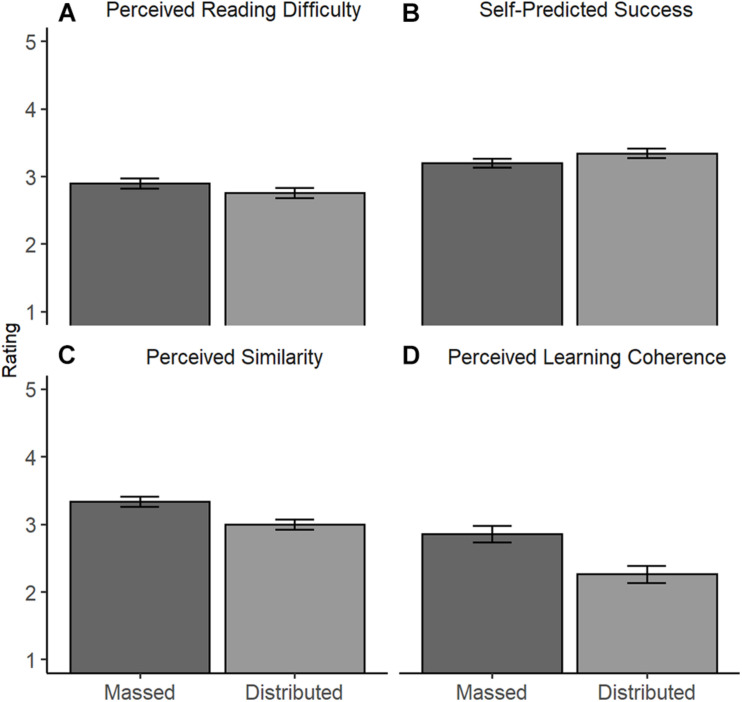
Main Effect of Learning Condition on Meta-Cognitive Judgments of Learning in Experiment 1. Estimated means of **(A)** perceived difficulty (recoded, 1: very easy; 5: very difficult), **(B)** predicted success (recoded, 1 = very good; 5 = very bad), **(C)** perceived similarity, and **(D)** perceived learning coherence (1 = not true; 5 = true) for the learning conditions, Experiment 1. Error bars represent standard errors (-/ + 1 SE).

The model revealed an effect of the learning condition, *F*(2,278) = 3.23, *p* = 0.041. However, despite the higher difficulty and lower predicted success predicted by Hypothesis 3, in the distributed condition, participants in the distributed condition perceived reading as less difficult (*M* = 2.75, *SE* = 0.07), but predicted lower success (*M* = 3.34, *SE* = 0.07) than participants in the in the massed condition (perceived reading difficulty: *M* = 2.90, *SE* = 0.07; predicted success: *M* = 3.20, *SE* = 0.07, Figure 3). Please note that the univariate tests failed to reach significance (for both tests: | *t|* > 1.32 and *p* > 0.065, one-tailed) even though the multivariate analysis supports Hypothesis 3.

We found no interaction between learning condition and text, *F*(2, 278) = 0.50, *p* = 0.606. Thus, participants in the distributed condition perceived both texts as more difficult than participants in the massed condition.

##### Meta-cognitive judgments of perceived similarity and learning coherence

The model revealed a significant effect of the learning condition *F*(2, 137) = 9.13, *p* < 0.001. In line with Hypothesis 4, participants in the distributed condition perceived less similarity and learning coherence between the texts (perceived similarity: *M* = 3.00, *SE* = 0.08; learning coherence: *M* = 2.26, *SE* = 0.12) than participants in the massed condition [perceived similarity: *M* = 3.34, *SE* = 0.08, *t*(138) = -3.11, *p* = 0.002; learning coherence: *M* = 2.85, *SE* = 0.12, *t*(138) = -3.34, *p* = 0.001; Figure 3].

### Discussion

In Experiment 1, learning outcomes were not enhanced by distributed learning, not even at the longer retention interval. Nevertheless, learning outcomes decreased from the short- to the long-retention interval only in the massed but not in the distributed condition. Consequently, learning outcomes in the massed and the distributed condition were on par 1 week after the second text had been read. On the one hand, one interpretation of this pattern of effects is that distributed reading made the learning outcomes more stable. On the other hand, given that we did not find a benefit of distributed reading and the performance in both conditions was very low in both groups, this pattern might also be the result of a bottom effect in the distributed condition. Thus, distributed learning might have been too difficult for participants to be desirable for long-term learning. Furthermore, it might be argued that distributed learning is confounded with the retention interval. Comprehension questions referred to Text 1, Text 2, or both texts. As participants read Text 1 1 week earlier in the distributed condition, the short retention interval for questions that referred to this text was actually not 5 min but 1 week.

Consistent with previous studies, Experiment 1 showed that learning from text increased with higher levels of domain-specific prior knowledge (e.g., Schneider et al., 1989; Ozuru et al., 2009). The results regarding the meta-cognitive judgments of predicted success and reading difficulty were somewhat mixed, but seem to indicate that distributed learning changes the perceived difficulty in learning, with lower perceived difficulty during reading but, at the same time, lower predicted success of learning. Furthermore, the meta-cognitive judgments of perceived similarity and learning coherence suggest that distributed learning made coherence-building across texts subjectively more difficult for the learner, possibly by making the retrieval of information from the first text more effortful.

The main weakness of Experiment 1 is the potential confound between learning condition and retention interval. This confound is a design feature of distributed learning and introduces some ambiguity into the interpretation of results. One way to preserve the potential benefits of distributed reading and simultaneously eliminate the potential drawbacks is to use a shorter interval between the two texts. This possibility was explored in Experiment 2.

## Experiment 2

Studies on the benefits of the retrieval practice effect have shown that retrieval success is essential for long-term retention. Thus, the low, albeit stable level of learning outcomes in the distributed condition might indicate that the interstudy interval of 1 week chosen in Experiment 1 was too long to enable successful retrieval of information from the first text. Therefore, we changed the lag between the two texts from 1 week to 15 min in Experiment 2. This relatively short lag might encourage active retrieval of the first text when reading the second text. The same hypotheses were tested as in Experiment 1. As in Experiment 1, we tested our hypotheses simultaneously with two different sets of texts from two domains.

### Method

#### Design

Experiment 2 was based on a 2 × 2 × 2 design with the independent variables learning condition (massed vs. distributed learning), retention interval (immediate vs. 1-week delay), and domain (biology vs. physics). Learning condition and domain were varied between participants, and retention interval was varied within participants. Participants were randomly assigned to one of the four resulting experimental groups. To minimize differences in learner characteristics between the experimental groups as in Experiment 1, we first formed homogeneous blocks of participants for each class matched according to prior knowledge and reading ability and then assigned participants from these groups randomly to the experimental conditions (randomized block design) within classes. All experimental conditions were realized in each class. The assignment of the two comprehension tests to the two levels of the factor retention interval was counterbalanced between participants.

#### Participants

Participants in Experiment 2 were 160 seventh graders (77 boys, 83 girls), with a mean age of 12.97 (*SD* = 0.44) from eight classes of different schools (Gymnasium and comprehensive schools). For all participating participants, parental permission was obtained. Participants without parental permission solved riddles instead of participating in the experiments (alternatively, they were allowed to visit parallel classes). As in Experiment 1, participants were randomly assigned to massed (*n* = 81) and the distributed condition (*n* = 79). Participants received sweets and a magic cube puzzle as a reward for participation.

Fourteen participants missed one of the two learning sessions and were excluded from analysis. Thirteen participants missed the domain-specific prior knowledge test, their data was excluded from analysis. The data of two participants was excluded because of technical problems, and one participant could not complete the experiment because of language issues. In the end, the data of 130 participants were analyzed (68 in the massed and 62 in the distributed learning condition).

#### Power Analysis

As for Experiment 1, we estimated the power for a small interaction effect between learning condition and retention interval by simulation using the R package simr (Green and MacLeod, 2016). The power (1-β) for detecting a small effect (*d* = 0.3 or OR = 1.72) was high (1.000, 95% CI [0.996, 1.000]) given the Type-I error probability (α) of 0.05.

#### Materials

The materials of Experiment 1 were used for Experiment 2 with slight changes. The low learning outcomes in Experiment 1 might be due to the fact that the texts were too difficult. Therefore, we revised the experimental texts by implementing more examples and illustrative metaphors. The Flesch reading ease changed only moderately (Biology 1: 57, Biology 2: 46, Physics 1: 43, Physics 2: 57).

The comprehension tests (two for each domain with multiple-choice and short-answer questions) remained unchanged (Cohen’s κ = 84). The mean item difficulty was 0.33 (*SD* = 0.20) for the short-answer questions and 0.52 (*SD* = 0.13) for the multiple-choice items.

Reading ability (Cronbach’s α = 0.75), knowledge about reading strategies, and working memory capacity (Cronbach’s α = 0.85) were assessed with the same measures as in Experiment 1. In some classes, the RSPAN was aborted because of time issues. Therefore, for all participants, only the performance of the first 10 sequences was included in the analysis. In the prior knowledge test, one question from the physics part was dropped to equal the time spent on the physics test and the biology test (shortened physics test: Cohen’s κ = 0.86).

Unlike Experiment 1, metacognitive judgments of reading difficulty and predicted learning success were provided only after reading the second text to create a massed condition without any disruption. The ratings of perceived similarity (Cronbach’s α = 0.30) and learning coherence remained unchanged. The time allotted for the metacognitive judgments was set at 5 min, and filler questions were added at the end to ensure that all participants received the immediate test exactly 5 min after learning.

#### Procedure

**FIGURE 4:**
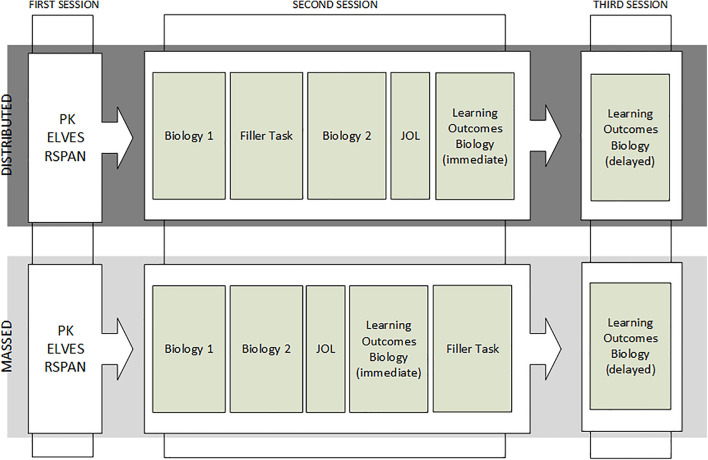
Procedure of Experiment 2 in the Biology Group. An illustration of the procedure of Experiment 2 in the biology group. The example was drawn from the biology group, the procedure was the same for the physics group with the respective texts for physics. PK = Assessment of prior knowledge; ELVES = Assessment of reading ability; RSPAN = Assessment of working memory; Biology 1 = first text biology [first text phyiscs]; JOL = assessment of meta-cognitive judgments of the learning process; Biology 2 = second text biology [second text physics]; and Learning outcomes biology = assessment of learning outcome for biology [assessment of learning outcome for physics]. Learning outcomes were measured immediate after learning (immediate) and 1 week delayed (delayed).

The procedure of Experiment 2 matched that of Experiment 1 with slight changes (Figure 4). After the pretest in Session 1, the experimental groups were matched by prior knowledge and reading ability.

In Session 2, participants read the two texts. In the distributed condition, reading was interrupted by a 15-min filler task between the first and second text, whereas in the massed condition, participants read the second text immediately after the first text without an intervening task. After reading the second text, the participants completed the assessment of the meta-cognitive judgments of the learning process, followed by the immediate assessment of learning outcomes.

The delayed assessment of learning outcomes was administered in Session 3, which was originally planned for 1 week after Session 1. However, this session was rescheduled in two classes. Thus, the length of the retention interval varied between 7 and 9 days.

### Results

#### Differences Between Experimental Groups

No differences were found between the learning conditions in the teacher reported grades of the participants in biology (*W* = 438, *p* = 0.410), but slight differences were found in the physics grades (*W* = 506.5, *p* = 0.047; massed: *Min* = 1, *Q1* = 1, *Mdn* = 2, *Q3* = 2, *Max* = 3; distributed: *Min* = 1, *Q1* = 2, *Mdn* = 2, *Q3* = 3, *Max* = 4). Also, no differences in working memory capacity and reading ability were found between the learning conditions [working memory: *F*(1,116) = 0.07, *p* = 0.788; reading ability: *F*(1,126) = 0.11, *p* = 0.738]. Additionally, no differences were found between the groups receiving the biology or the physics texts [working memory *F*(1,116) = 1.61, *p* = 0.207; reading ability: *F*(1,126) = 0.77, *p* = 0.381], indicating that the matching procedure was effective.

Table 3 provides the means and standard deviations of all dependent variables and learner characteristics observed in the four experimental groups. A correlation matrix (including the correlations within learning conditions/topics) of all dependent variables is provided in Supplementary Appendix Table B2.

**TABLE 3:** Mean and standard deviation of dependent variables and learner characteristics in experiment 2.

	Biology				Physics			
	Massed		Distributed		Massed		Distributed	
Variable	*M*	*SD*	*M*	*SD*	*M*	*SD*	*M*	*SD*
Dependent variables								
Learning outcome (immediate)	6.41	3.28	6.37	3.85	6.50	2.77	5.12	2.81
Learning outcome (delayed)	5.67	2.97	6.21	3.79	5.41	3.04	5.04	2.41
Perceived difficulty	3.74	0.75	3.33	0.99	3.38	0.85	3.38	0.91
Self-predicted success	2.91	0.57	2.53	0.97	2.79	0.54	2.81	0.78
Perceived similarity	3.39	0.63	3.57	0.68	3.24	0.66	3.24	0.42
Perceived learning coherence	2.76	0.89	2.57	1.10	2.82	1.00	2.56	1.22
**Learner characteristics**								
Domain-specific prior knowledge	1.97	1.85	2.93	2.32	6.24	2.10	5.19	2.26
Grades	2.31	0.89	2.48	0.89	1.82	0.77	2.29	0.85
Reading ability	15.22	3.76	16.10	6.06	17.12	5.99	15.66	4.27
Working memory capacity	0.66	0.18	0.71	0.19	0.76	0.13	0.69	0.17

*All variables are reported separately for the domains as domain was varied between-subjects.*

#### Learning Outcomes

To test Hypotheses 1 and 2, we estimated a generalized mixed model with learning condition (contrast-coded: distributed = 1, massed = -1) and retention interval (contrast-coded: immediate = -1, delayed = 1) with their interaction and domain-specific prior knowledge (*z*-transformed) as predictors. Text comprehension performance served as the dependent variable. In addition, we included domain3 (contrast-coded: physics = 1, biology = -1) as predictor for control purposes. Items and participants were included as random effects (random intercepts). The parameter estimates are provided in Table 4.

**TABLE 4:** Parameter estimates and significance tests for the generalized mixed model for learning outcomes in experiment 2.

Fixed effects		
**Predictors**	**Estimate**	***SE***
(Intercept)	−0.65***	0.16
Learning condition	−0.05	0.09
Retention interval	−0.13**	0.04
Prior knowledge (*z*-standardized)	0.61***	0.11
Domain	−0.46**	0.17
Learning condition x Retention interval	0.08^+^	0.04

**Random effects**		

σ^2^	3.29	
τ_00_	0.32 _*ID*_	
	1.43 _*Item*_	

**Goodness of fit**		

Deviance	4157.23	

*Learning condition (contrast-coded: distributed = 1, massed = -1). Retention interval (contrast-coded: immediate = -1, delayed = 1). Domain (contrast-coded: biology = -1, physics = 1). Prior knowledge was included *z*-standardized. Directional hypotheses were tested one-tailed.*

**p < 0.01 (two-tailed). ***p < 0.001 (two-tailed).

*^+^*p* < 0.05 (one-tailed).*

**FIGURE 5:**
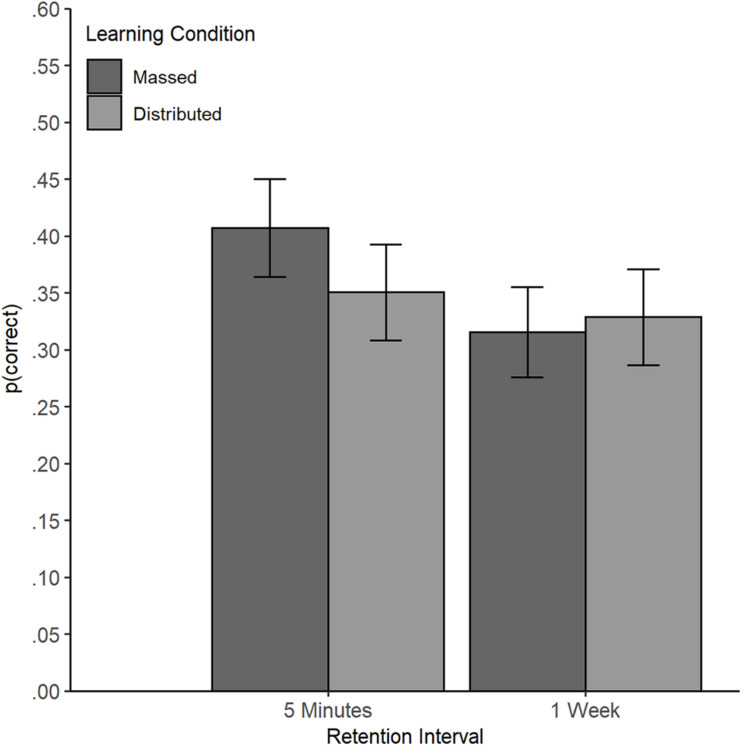
Interaction Between Learning Condition and Retention Interval in Experiment 2. Learning outcomes (text comprehension) by learning condition at the short and long retention interval in Experiment 2 [estimates based on Model 1, back-transformed probability (p) of a correct answer]. Error bars represent standard errors (±1 SE).

Paralleling the results of Experiment 1, there was no main effect of learning condition (β = -0.05, *SE* = 0.09, *z* = -0.54, and *p* = 0.588). Participants in the massed condition (probability = 0.36, *SE* = 0.04) performed equally well as participants in the distributed condition (probability = 0.34, *SE* = 0.04), *OR* = 1.10 (95% CI [0.78, 1.55]). The model revealed a significant main effect of retention interval (β = -0.13, *SE* = 0.04, *z* = -3.19, *p* = 0.001), with better performance at the short retention interval (probability = 0.38, *SE* = 0.04) compared to the long retention interval (probability = 0.32, *SE* = 0.04), *OR* = 0.77 (95% CI [0.66, 0.91]). Again, this main effect of retention interval was qualified by an interaction with the learning condition (β = 0.08, *SE* = 0.04, *z* = 1.95, *p* = 0.025, one-tailed, Figure 5), as predicted in Hypothesis 1. Planned comparisons revealed that the performance of participants in the massed condition decreased between the two retention tests (immediate test: probability = 0.41, *SE* = 0.04; delayed test: probability = 0.32, *SE* = 0.04), *z* = 3.79, *p* < 0.001, *OR* = 0.66 (95% CI [0.53, 0.82]). In contrast, performance in the distributed condition did not decrease significantly (immediate test: probability = 0.35, *SE* = 0.04; delayed test: probability = 0.33, *SE* = 0.04), *z* = 0.84, *p* = 0.399, *OR* = 0.90 (95% CI [0.71, 1.14]). Contrary to Experiment 1, we found no benefit of massed learning in the test immediately after reading (*z* = -1.35, *p* = 0.179), *OR* = 1.29 (95% CI [0.89, 1.86]). However, even though the participants in the distributed condition showed no decrease, they did not perform better than the participants in the massed condition at the delayed test (*z* = 0.32, *p* = 0.746), *OR* = 0.94 (95% CI [0.64, 1.38]).

Consistent with the findings in Experiment 1 and Hypothesis 2, we found a significant positive effect of prior knowledge (β = 0.61, *SE* = 0.11, *z* = 5.65, *p* < 0.001). A difference of one standard deviation in prior knowledge corresponded to an odds ratio of 1.84 (95% CI [1.49, 2.27]).

In sum, we did not find the benefit of distributed reading for lasting learning that was predicted in Hypothesis 1. However, we found no detrimental effects of distributed reading at the immediate but still no decrease in the distributed condition between the immediate and the delayed test. Hypothesis 2 stating that domain-specific prior knowledge would benefit learning from the texts was again supported by the data.

#### Metacognitive Judgments of the Learning Process

We estimated two multivariate linear models with the metacognitive judgments as dependent variables and learning condition (contrast-coded: distributed = 1, massed = -1), *z*-standardized prior knowledge and their interaction, and domain (contrast-coded: biology = -1, physics = 1) as predictors. In both models, no significant main effects of learning condition were found [predicted success and reading difficulty: *F*(2,126) = 0.93, *p* = 0.397; perceived similarity and learning coherence: *F*(2,126) = 1.01, *p* = 0.367].

### Discussion

Experiment 2 replicated the main findings of Experiment 1 but with a much shorter interstudy interval (15 min instead of 1 week). Distributed learning did not lead to better learning results, not even at the delayed test. Nevertheless, students in the distributed condition showed no decrease in learning outcome from the immediate to the delayed test, whereas performance of students in the massed condition did. This result is especially noteworthy because both experimental groups performed equally well at the test immediately after learning. Thus, contrary to Experiment 1, the fact that the learning outcomes in the distributed condition did not decrease from the immediate to the delayed test cannot be attributed to disadvantages due to a longer retention interval or a floor effect at the immediate test.

Contrary to the findings of Experiment 1, distributed learning was not associated with higher perceived difficulty and predicted success. Furthermore, no differences were found in perceived learning coherence and similarity between the learning conditions. Apparently, the 15 min interstudy interval in the distributed learning condition was not sufficiently to make learning subjectively more difficult. Nevertheless, distributed learning made a difference for the learning outcomes by slowing down the decrease in learning from the immediate test to the delayed test after 1 week.

## General Discussion

The present experiments addressed the question of whether distributed learning is beneficial for learning with multiple, complementary texts, especially in the long-term. The experiments were conducted with seventh graders who read two expository texts from the two domains of physics and biology. Experiment 1 implemented a long interstudy interval (1 week), whereas Experiment 2 implemented a short interstudy interval (15 min). Both experiments showed a highly similar pattern of learning outcomes. Learning outcomes decreased from the immediate to the delayed test in massed but not in distributed reading. Nevertheless, participants who had read the texts in a distributed fashion performed no better than participants in the massed condition at the delayed test. Moreover, the results for metacognitive judgments show that participants in Experiment 1 perceived the text in distributed reading as less difficult and predicted that they would be less successful in recalling information from this text, and they perceived the two texts as less coherent and less similar compared to massed reading. This could indicate that the participants recognized the lower learning coherence and anticipated the detrimental effects on learning in the immediate test, but did not use these metacognitive judgments to engage more in reading, what might have been reflected in higher perceived difficulty and better learning outcomes. In contrast, no such effects occurred in Experiment 2 with the short interstudy interval of 15 min.

### Inhibitors of Advantages of Distributed Learning

Overall, we found no support for our assumption that distributed reading benefits long-term retention. Given that distributed practice is effective even without repetition, some features of our experiments, or of distributed reading in general, might have reduced the benefits of distribution. First of all, long-term retention was measured 1 week after learning, which is a relatively short retention interval according to prior research that suggests retention intervals of 4 weeks or longer (Rohrer, 2015). As mentioned above, desirable difficulties might play out their advantages in particular at longer intervals between learning and assessment of learning outcomes (Rawson and Kintsch, 2005; Pashler et al., 2007; Rawson, 2012, see also Dunlosky et al., 2013). This might be also the case for distributed reading.

Another feature that might have reduced the benefits of distribution, is that we varied the retention interval within subjects. However, immediate test without feedback facilitates long-term learning more than a delayed retention test, provided that retrieval is successful (Karpicke and Roediger, 2010; Greving and Richter, 2018). If such a testing effect occurred in the present experiments, students in the massed condition might have been advantaged, especially in Experiment 1, because of their better recall success at the immediate test. This advantage might have led to an underestimation of the benefits of distributed reading. In future research, a between-subjects variation of the retention interval should be considered.

Additionally, distributed reading lacks (in)direct feedback. Research in retrieval practice has shown that feedback is crucial for retrieval practice effects with short-answer questions and after an incorrect response (Pashler et al., 2005; Kang et al., 2007). In distributed practice, the repetition of learning material provides not only an additional learning occasion but also serves as a kind of feedback for the retrieval of the first learning session. This indirect feedback might be essential to the benefits of distributed learning. For example, in the study by Vlach and Sandhofer (2012), the concept of food chains was repeated in each biome. Thus, even if the children failed to remember parts of the food chain of the biome explained in the last session, they had the opportunity to update the knowledge and then use the repeated presentation of the concepts as feedback for their retrieval and overall comprehension. However, in distributed reading, no such indirect feedback is provided.

An important distinction of our study is that we investigated distributed learning in a field setting with younger learners. The distributed practice effect is a robust finding with adult learners in laboratory settings, but few studies have investigated the extent that these findings generalize to younger learners and to learning in real-world educational settings (Küpper-Tetzel, 2014). However, in school contexts, even distributed practice might fail to benefit learning, for example due to a noisier learning environment (Goossens et al., 2016). A recent study that investigated the effects of rereading schedules in the same age groups arrived at conclusions very similar to those of the present study (Greving and Richter, 2019). In this study, seventh graders read a text about a bacterial cell twice. Rereading was implemented either in a massed fashion, with no interruption between reading and rereading, or in a distributed fashion, with a lag of 1 week between reading and rereading. Students rereading the texts in a distributed fashion predicted their recall to be lower, and indeed they showed lower recall and text comprehension performance, but only immediately after reading. 1 week later, no difference was found between the massed and distributed condition. Moreover, a lower decrease between tests occurred in the distributed condition. The performance remained stable in the distributed condition, but the performance between the immediate and delayed post-test decreased in the massed condition. However, distributed rereading appears to have no advantage over massed rereading even after a retention interval of 1 week. Given the positive results for distributed rereading with adults (e.g., Rawson and Kintsch, 2005), distributed rereading might be less effective for younger learners than for adult learners, possibly because of the lower comprehension skills of younger learners. A similar relationship might hold for distributed reading, as investigated in the present experiments. Thus, research with adult learners seems to be necessary to evaluate the effectiveness of distributed reading.

### Limitations and Suggestions for Further Research

The results of the two experiments are consistent but need to be interpreted with a number of limitations in mind. One limitation of the research reported here is that we were able to examine the effects of only two lags, which differed considerably in length. In Experiment 1, we tried to implement an “authentic” lag of 1 week, which is quite typical for school environments with a weekly schedule. However, this design decision caused a potential confound of distributed vs. massed reading with the time between reading the first text and the retention test in the distributed condition. We implemented a lag that diminishes this confounding variable in Experiment 2 by choosing a lag that was in line with earlier research on rereading with very short lags (Glover and Corkill, 1987). However, the school setting put constraints on the length of this lag, and 15 min was the maximal lag we could implement within one session. The end result could have been that the long lag implemented in Experiment 1 was too long, whereas the short lag implemented in Experiment 2 was too short to produce beneficial effects of distributed reading. Further research should explore a wider range of lags between 15 min and 1 week.

A second limitation is that the retention interval of 1 week used in our experiments might have been too short to detect an advantage of distributed reading. A study with university students that was otherwise very similar to the present experiments used a 2-week retention interval between the final learning session and the assessment of learning outcomes, with comparable results and still no evidence for an advantage of distributed over massed reading at the long retention interval (Greving and Richter, 2021). However, apart from the fact that university students differ in many ways from secondary school students, a systematic manipulation of retention intervals, including also intervals of several weeks, would be needed.

Finally, although all efforts were made to maximize the ecological validity of the study, for example by selecting a topic from the curriculum and typical expository texts, the learning situation still deviated in several respects from normal school learning. In particular, the individual results in the learning outcome measure did not count for students’ grades. Thus, the study combined a highly demanding learning task with a low-stakes assessment, which is unusual in school learning and may have caused the students not to take the learning task seriously enough. The low accuracy in the learning outcomes measure suggests such an interpretation.

### Conclusion

Despite these limitations, some cautious implications can be drawn. The distribution of learning episodes is often recommended as general learning strategy (Schwartz et al., 2011; Putnam et al., 2016). Refining this general recommendation, our research suggests that teachers who wish to use distribution of learning as didactic strategy should restrict the usage to materials which include at least some kind of repetition.

This research was the first to investigate distributed learning with multiple complementary texts. We found evidence that distributed learning might change memory, indicated by lower decrease between immediate and delayed test, but contrary to our expectations, it did not lead to better learning outcomes at a retention interval of 1 week. These results have practical import. Given well-established benefits of distributed practice, students are often advised to space their learning activities rather than massing them in one learning session. However, this advice is not always restricted to repetitive learning. As noted above, Schwartz et al. (2011, p. 10) defined distributed practice as “learning that is spread out across relatively long periods of time rather than massed all at once.” Similarly, in their study guide for college students, Putnam et al. (2016) give students the recommendation to engage in distributed learning because “by spacing your studying you will learn the material in less time than if you tried to cram all of your studying into the night before the test” (p. 655). But is distributed practice a good strategy even for non-repeated materials? To date, the research on distributed practice cannot provide a clear answer to this question. It seems that more research on distributed learning with complementary learning materials is needed before it can be recommended as an effective learning strategy to students and teachers. The research reported here can be seen as a first step in this direction.

## Data Availability Statement

The datasets presented in this study can be found in online repositories. The names of the repository/repositories and accession number(s) can be found below: OSF; https://osf.io/z8aqn/.

## Ethics Statement

Ethical review and approval was not required for the study on human participants in accordance with the local legislation and institutional requirements. Written informed consent to participate in this study was provided by the participants’ legal guardian/next of kin.

## Author Contributions

CEG designed the research, organized the experiment conduction, analyzed and interpreted the data, and wrote the article. TR supervised the project, designed the research, and revised the article. Both authors contributed to the article and approved the submitted version.

## Conflict of Interest

The authors declare that the research was conducted in the absence of any commercial or financial relationships that could be construed as a potential conflict of interest.

## Publisher’s Note

All claims expressed in this article are solely those of the authors and do not necessarily represent those of their affiliated organizations, or those of the publisher, the editors and the reviewers. Any product that may be evaluated in this article, or claim that may be made by its manufacturer, is not guaranteed or endorsed by the publisher.

## References

[B1] Alexander P. A. Jetton T. L. (2003). “Learning from traditional and alternative texts: new conceptualizations for the information age,” in *Handbook of Discourse Processes*, eds Graesser A. C. Gernsbacher M. A. Goldman S. R. (New York, NY: Routledge), 199–242.

[B2] Alter A. L. Oppenheimer D. M. (2009). Uniting the tribes of fluency to form a metacognitive nation. *Personal. Soc. Psychol. Rev.* 13 219–235. 10.1177/1088868309341564 19638628

[B3] Amstad T. (1978). *Wie Verständlich Sind Unsere ZEITUNGEN? [How Understandable are our Newspapers?].* Zürich: Studenten-Schreib-Service.

[B4] Baayen R. H. Davidson D. J. Bates D. M. (2008). Mixed-effects modeling with crossed random effects for subjects and items. *J. Mem. Lang.* 59 390–412. 10.1016/j.jml.2007.12.005

[B5] Barr D. J. Levy R. Scheepers C. Tily H. J. (2013). Random effects structure for confirmatory hypothesis testing: keep it maximal. *J. Mem. Lang.* 68 255–278. 10.1016/j.jml.2012.11.001 24403724 PMC3881361

[B6] Bates D. Mächler M. Bolker B. Walker S. (2015). Fitting linear mixed-effects models using lme4. *J. Stat. Softw.* 67 1–48. 10.18637/jss.v067.i01

[B7] Bjork E. L. Bjork R. A. (2011). “Making things hard on yourself, but in a good way: creating desirable difficulties to enhance learning,” in *Psychology and the Real World: Essays Illustrating Fundamental Contributions to Society*, eds Gernsbacher M. A. Pew R. W. Hough L. M. Pomerantz J. R. (New York, NY: Worth Publishers), 56–64.

[B8] Bjork R. A. (1994). “Memory and metamemory considerations in the training of human beings,” in *Metacognition: Knowing About Knowing*, eds Metcalfe J. Shimamura A. (Cambridge: MA: MIT Press), 185–205.

[B9] Bjork R. A. Dunlosky J. Kornell N. (2013). Self-regulated learning: beliefs, techniques, and illusions. *Annu. Rev. Psychol.* 64 417–444. 10.1146/annurev-psych-113011-143823 23020639

[B10] Boscolo P. Mason L. (2003). Topic knowledge, text coherence, and interest: how they interact in learning from instructional texts. *J. Exp. Educ.* 71 126–148.

[B11] Braun K. Rubin D. C. (1998). The spacing effect depends on an encoding deficit, retrieval, and time in working memory: evidence. *Memory* 6 37–66. 10.1080/741941599 9640432

[B12] Carpenter S. K. Cepeda N. J. Rohrer D. Kang S. H. K. Pashler H. (2012). Using spacing to enhance diverse forms of learning: review of recent research and implications for instruction. *Educ. Psychol. Rev.* 24 369–378. 10.1007/s10648-012-9205-z

[B13] Cepeda N. J. Coburn N. Rohrer D. Wixted J. T. Mozer M. C. Pashler H. (2009). Optimizing distributed practice: theoretical analysis and practical implications. *Exp. Psychol.* 56 236–246. 10.1027/1618-3169.56.4.236 19439395

[B14] Cepeda N. J. Pashler H. Vul E. Wixted J. T. Rohrer D. (2006). Distributed practice in verbal recall tasks: a review and quantitative synthesis. *Psychol. Bull.* 132 354–380. 10.1037/0033-2909.132.3.354 16719566

[B15] Cepeda N. J. Vul E. Rohrer D. Wixted J. T. Pashler H. (2008). Spacing effects in learning a temporal ridgeline of optimal retention. *Psychol. Sci.* 19 1095–1102. 10.1111/j.1467-9280.2008.02209.x 19076480

[B16] Cohen J. (1988). *Statistical Power Analysis for the Behavioral Sciences*, 2nd Edn. Hillsdale, NJ: L. Erlbaum Associates.

[B17] Delaney P. F. Verkoeijen P. P. J. L. Spirgel A. (2010). “Spacing and testing effects: a deeply critical, lengthy, and at times discursive review of the literature,” in *Psychology of Learning and Motivation: Advances in Research and Theory*, ed. Ross B. (San Diego, CA: Elsevier Academic Press), 63–147. 10.1016/S0079-7421(10)53003-2

[B18] Dempster F. N. (1989). Spacing effects and their implications for theory and practice. *Educ. Psychol. Rev.* 1 309–330. 10.1007/BF01320097

[B19] Dixon P. (2008). Models of accuracy in repeated-measures design. *J. Mem. Lang.* 59 447–456. 10.1016/j.jml.2007.11.004

[B20] Donovan J. J. Radosevich D. J. (1999). A meta-analytic review of the distribution of practice effect: now you see it, now you don’t. *J. Appl. Psychol.* 84 795–805. 10.1037/0021-9010.84.5.795

[B21] Dunlosky J. Rawson K. A. Marsh E. J. Nathan M. J. Willingham D. T. (2013). Improving students’ learning with effective learning techniques: promising directions from cognitive and educational psychology. *Psychol. Sci. Public Interest* 14 4–58. 10.1177/1529100612453266 26173288

[B22] Feng S. D’Mello S. Graesser A. C. (2013). Mind wandering while reading easy and difficult texts. *Psychon. Bull. Rev.* 20 586–592. 10.3758/s13423-012-0367-y 23288660

[B23] Fox J. Weisberg S. (2019). *An R Companion to Applied Regression*, 3 Edn. Thousand Oaks CA: Sage.

[B24] Glover J. A. Corkill A. J. (1987). Influence of paraphrased repetitions on the spacing effect. *J. Educ. Psychol.* 79 198–199. 10.1037/0022-0663.79.2.198

[B25] Goossens N. A. M. C. Camp G. Verkoeijen P. P. J. L. Tabbers H. K. Bouwmeester S. Zwaan R. A. (2016). Distributed practice and retrieval practice in primary school vocabulary learning: a multi-classroom study: distributed practice and retrieval practice. *Appl. Cogn. Psychol.* 30 700–712. 10.1002/acp.3245

[B26] Green P. MacLeod C. J. (2016). simr?: an R package for power analysis of generalized linear mixed models by simulation. *Methods Ecol. Evol.* 7 493–498. 10.1111/2041-210X.12504

[B27] Greving C. E. Richter T. (2019). Distributed learning in the classroom: effects of rereading schedules depend on time of test. *Front. Psychol.* 9:2517. 10.3389/fpsyg.2018.02517 30687145 PMC6333692

[B28] Greving C. E. Richter T. (2021). *Learning from Complementary Texts: is Distributed Reading a Desirable Difficulty?* (Manuscript submitted for publication).

[B29] Greving S. Richter T. (2018). Examining the testing effect in university teaching: retrievability and question format matter. *Front. Psychol.* 9:2412. 10.3389/fpsyg.2018.02412 30564174 PMC6288371

[B30] Inquisit 3 (Version 3.0.6.0) (2011). *Precision Software for Cognitive, Social, Neurophysiological, and Online Psychological Experiments. [Computer software]*. Available online at: http://www.millisecond.com (accessed March, 2015).

[B31] Jaeger T. F. (2008). Categorical data analysis: away from ANOVAs (transformation or not) and towards logit mixed models. *J. Mem. Lang.* 59 434–446. 10.1016/j.jml.2007.11.007 19884961 PMC2613284

[B32] Kang S. H. K. McDermott K. B. Roediger H. L. (2007). Test format and corrective feedback modify the effect of testing on long-term retention. *Eur. J. Cogn. Psychol.* 19 528–558. 10.1080/09541440601056620

[B33] Karpicke J. D. Roediger H. L. (2010). Is expanding retrieval a superior method for learning text materials? *Mem. Cognit.* 38 116–124. 10.3758/MC.38.1.116 19966244

[B34] Kintsch W. (1988). The role of knowledge in discourse comprehension: a construction-integration model. *Psychol. Rev.* 95 163–182. 10.1037/0033-295X.95.2.163 3375398

[B35] Kintsch W. (1994). Text comprehension, memory, and learning. *Am. Psychol.* 49 294–303. 10.1037/0003-066X.49.4.294 8203801

[B36] Kornell N. (2009). Optimising learning using flashcards: spacing is more effective than cramming. *Appl. Cogn. Psychol.* 23 1297–1317. 10.1002/acp.1537

[B37] Küpper-Tetzel C. E. (2014). Strong effects on weak theoretical grounds: understanding the distributed practice effect. *Z. Für Psychol.* 222 71–81. 10.1027/2151-2604/a000168

[B38] Kuznetsova A. Brockhoff P. B. Christensen R. H. B. (2017). lmerTest package: tests in linear mixed effects models. *J. Stat. Softw.* 82 1–26. 10.18637/jss.v082.i13

[B39] Lenth R. V. (2016). Least-squares means: the R package lsmeans. *J. Stat. Softw.* 69 1–33. 10.18637/jss.v069.i01

[B40] Lipowsky F. Richter T. Borromeo-Ferri R. Ebersbach M. Hänze M. (2015). Wünschenswerte erschwernisse beim Lernen [Desirable difficulties in learning]. *Schulpädag. Heute* 6 1–10.

[B41] Matuschek H. Kliegl R. Vasishth S. Baayen H. Bates D. (2017). Balancing Type I error and power in linear mixed models. *J. Mem. Lang.* 94 305–315. 10.1016/j.jml.2017.01.001

[B42] Mayer R. E. (1996). Learners as information processors: legacies and limitations of educational psychology’s second metaphor. *Educ. Psychol.* 31 151–161. 10.1080/00461520.1996.9653263

[B43] McCrudden M. Schraw G. Hartley K. Kenneth A. K. (2004). The influence of presentation, organization, and example context on text learning. *J. Exp. Educ.* 72 289–306. 10.3200/JEXE.72.4.289-306

[B44] McDaniel M. A. Hines R. J. Guynn M. J. (2002). When text difficulty benefits less-skilled readers. *J. Mem. Lang.* 46 544–561. 10.1006/jmla.2001.2819

[B45] McNamara D. S. (2001). Reading both high-coherence and low-coherence texts: effects of text sequence and prior knowledge. *Can. J. Exp. Psychol. Can. Psychol. Exp.* 55 51–62. 10.1037/h0087352 11301728

[B46] McNamara D. S. Kintsch W. (1996). Learning from texts: effects of prior knowledge and text coherence. *Discourse Process.* 22 247–288. 10.1080/01638539609544975

[B47] McNamara D. S. Kintsch E. Songer N. B. Kintsch W. (1996). Are good texts always better? Interactions of text coherence, background knowledge, and levels of understanding in learning from text. *Cogn. Instr.* 14 1–43. 10.1207/s1532690xci1401_1

[B48] Mills C. D’Mello S. K. Kopp K. (2015). The influence of consequence value and text difficulty on affect, attention, and learning while reading instructional texts. *Learn. Instr.* 40 9–20. 10.1016/j.learninstruc.2015.07.003

[B49] Oberauer K. Süß H.-M. Schulze R. Wilhelm O. Wittmann W. W. (2000). Working memory capacity—facets of a cognitive ability construct. *Personal. Individ. Differ.* 29 1017–1045. 10.1016/S0191-8869(99)00251-2

[B50] Ozuru Y. Dempsey K. McNamara D. S. (2009). Prior knowledge, reading skill, and text cohesion in the comprehension of science texts. *Learn. Instr.* 19 228–242. 10.1016/j.learninstruc.2008.04.003

[B51] Pashler H. Cepeda N. J. Wixted J. T. Rohrer D. (2005). When does feedback facilitate learning of words? *J. Exp. Psychol. Learn. Mem. Cogn*. 31 3–8. 10.1037/0278-7393.31.1.3 15641900

[B52] Pashler H. Rohrer D. Cepeda N. J. Carpenter S. K. (2007). Enhancing learning and retarding forgetting: choices and consequences. *Psychonom. Bull. Rev.* 14 187–193. 10.3758/BF03194050 17694899

[B53] Primor L. Katzir T. (2018). Measuring multiple text integration: a review. *Front. Psychol.* 9:2294. 10.3389/fpsyg.2018.02294 30555372 PMC6282655

[B54] Putnam A. L. Sungkhasettee V. W. Roediger H. L. III (2016). Optimizing learning in college: tips from cognitive psychology. *Perspect. Psychol. Sci.* 11 652–660. 27694461 10.1177/1745691616645770

[B55] Randler C. Kranich K. Eisele M. (2008). Block scheduled versus traditional biology teaching—an educational experiment using the water lily. *Instr. Sci.* 36 17–25. 10.1007/s11251-007-9020-y

[B56] Rawson K. A. (2012). Why do rereading lag effects depend on test delay? *J. Mem. Lang.* 66 870–884. 10.1016/j.jml.2012.03.004

[B57] Rawson K. A. Kintsch W. (2005). Rereading effects depend on time of test. *J. Educ. Psychol.* 97 70–80. 10.1037/0022-0663.97.1.70

[B58] Richter T. (2006). What is wrong with anova and multiple regression? Analyzing sentence reading times with hierarchical linear models. *Discourse Process.* 41 221–250. 10.1207/s15326950dp4103_1

[B59] Richter T. van Holt N. (2005). ELVES: ein computergestütztes diagnostikum zur erfassung der effizienz von teilprozessen des leseverstehens [ELVES: a computer-based test for measuring efficiency of processes involved in reading comprehension]. *Diagnostica* 51 169–182. 10.1026/0012-1924.51.4.169

[B60] Richter T. Münchow H. Abendroth J. (2020). “The role of validation in integrating multiple perspectives,” in *Handbook of Learning from Multiple Representations and Perspectives*, eds Van Meter P. List A. Lombardi D. Kendeou P. (New York, NY?: Routledge), 259–275. 10.4324/9780429443961-18.

[B61] Rohrer D. (2015). Student instruction should be distributed over long time periods. *Educ. Psychol. Rev.* 27 635–643. 10.1007/s10648-015-9332-4

[B62] Schlagmüller M. Schneider W. (2007). *Würzburger Lesestrategie-Wissenstest für die Klassen 7-12 (WLST 7-12) [Würzburg Reading Strategy Knowledge Test for Grades 7-12].* Göttingen: Hogrefe.

[B63] Schmitt N. (1996). Uses and abuses of coefficient alpha. *Psychol. Assess.* 8 350–353. 10.1037/1040-3590.8.4.350

[B64] Schneider W. Korkel J. Weinert F. E. (1989). Domain-specific knowledge and memory performance: a comparison of high- and low-aptitude children. *J. Educ. Psychol.* 81 306–312. 10.1037/0022-0663.81.3.306

[B65] Schwartz B. L. Son L. K. Kornell N. Finn B. (2011). Four principles of memory improvement: a guide to improving learning efficiency. *Int. J. Creat. Probl. Solv.* 21 7–15.

[B66] Smith S. M. Rothkopf E. Z. (1984). Contextual enrichment and distribution of practice in the classroom. *Cogn. Instr.* 1 341–358. 10.1207/s1532690xci0103_4

[B67] Son L. K. Simon D. A. (2012). Distributed learning: data, metacognition, and educational implications. *Educ. Psychol. Rev.* 24 379–399. 10.1007/s10648-012-9206-y

[B68] Toppino T. C. Gerbier E. (2014). About practice: repetition, spacing and abstraction. *Psychol. Learn. Motiv.* 60 113–189.

[B69] van den Broek P. Risden K. Fletcher C. R. Thurlow R. (1996). “A ‘landscape’ view of reading: fluctuation patterns of activation and the construction of a stable memory representation,” in *Models of Understanding Text*, eds Britton B. K. Graesser A. C. (Mahwah, NJ: Lawrance Erlbaum Associates), 165–187.

[B70] Verkoeijen P. P. J. L. Rikers R. M. J. P. Özsoy B. (2008). Distributed rereading can hurt the spacing effect in text memory. *Appl. Cogn. Psychol.* 22 685–695. 10.1002/acp.1388

[B71] Vlach H. A. (2014). The spacing effect in children’s generalization of knowledge: allowing children time to forget promotes their ability to learn. *Child Dev. Perspect.* 8 163–168. 10.1111/cdep.12079

[B72] Vlach H. A. Sandhofer C. M. (2012). Distributing learning over time: the spacing effect in children’s acquisition and generalization of science concepts. *Child Dev.* 83 1137–1144. 10.1111/j.1467-8624.2012.01781.x 22616822 PMC3399982

[B73] Wittrock M. C. (2010). Learning as a generative process. *Educ. Psychol.* 45 40–45. 10.1080/00461520903433554

