# Supplementary material for: Beyond the Distributed Practice Effect: Is Distributed Learning Also Effective for Learning With Non-repeated Text Materials?
Source: Front Psychol. 2021 Oct 15;12:685245. doi: 10.3389/fpsyg.2021.685245 (PMC8554015; doi:10.3389/fpsyg.2021.685245)
Supplement: Supplementary file 1 [file Data_Sheet_1.docx]

Supplemental Materials “Beyond the Distributed Practice Effect: Is Distributed Learning Also Effective for Learning With Non-repeated Text Materials?”

# Appendix A

Text Materials Used in Experiment 1 (English Translations, the Original Texts are in German)

A.1 Biology

Text 1: Plant Cell

Page 1

The plant cell

Today, you will meet the plant cell. In the illustration, you can already see how the plant cell is structured.

As you may already know, cells are the basic components of all living beings. There are many different cells, which differ in shape, size, and function. In your body, there are 100,000,000,000,000 cells, including 210 different cells types that look different and fulfill different tasks. For example, liver cells are responsible for the detoxification of your body, and red blood cells provide your body with oxygen. Every cell in your body has a nucleus, the cell nucleus. All animal and plant cells also have a cell nucleus. Cells with a cell nucleus are called eukaryotes. However, some cells have no cell nucleus. Those cells are called prokaryotes. You will meet such a cell in the next chapter.

Page 2:

In this chapter, you will get to know the typical structure of a plant cell, which belongs to the eukaryotes.

If you examine, for example, a membrane of an onion under a microscope, you will see how the cells are separated by a thin wall. This wall is called the cell wall, which mainly consists of the most important building material of plants called cellulose. All plant cells are characterized by such a cell wall made of cellulose. The cell wall provides strength for the cell and separate the cell from the surrounding. You can see in the illustration, that the cell wall has some openings. These openings are called pits. At these places, substances can enter or leave the cell.

The interior of the cell is mostly filled with a liquid or gel-like mass called the cell plasma. This cell plasma is surrounded by a thin layer called the cell membrane.

Page 3

In the cell plasma, you can find the cell nucleus, which you already briefly encountered above. The cell nucleus is the carrier of the genetic information of the cell. Thus, it contains the “construction plan” for the entire cell. The cell nucleus is also surrounded by a membrane, which separates the cell nucleus from the cell plasma.

The cell plasma, of course, contains the cell nucleus but also different organelles, which means small organ. Organelles undertake independent tasks and thus are somewhat comparable with organs of multicellular organisms. One of those organelles is the Golgi apparatus, which is also called the “post office” of the cell. Here, substances are sorted, packed in small bubbles and sent to their destinations. One substance sorted by the Golgi apparatus is protein. Protein is produced by another organelle called a ribosome. Proteins can be used, for example, to build membranes.

Page 4

Cells also need a “power plant” to provide the cell with energy. For this task, another organelle is responsible, called the mitochondria.

The biggest cell organelles are the vacuoles. Vacuoles are small bubbles that merge over time into one big bubble. This bubble stores important nutrients and water for the cell.

One last organelle is the chloroplast, which is the solar plant of the cell in which photosynthesis takes place. Through photosynthesis, plant cells can produce energy from light.

In the illustration, you can see all components of the cell again.

Text 2: Bacterial cell

Page 1

The bacterial cell

In the last chapter, you already learned several facts about cells. Today, you will meet a new cell: the bacterial cell. In the illustration, you can already see how the bacterial cell is structured.

Many people only know bacteria from illnesses, for example, from infected tonsils. If you have such an illness, the doctor will most likely prescribe antibiotics. Antibiotics are a type of medication that kills disease-causing bacteria, which helps ill people get well again.

But not all bacteria are harmful. Many useful bacteria exist in the world that clean water and decompose organic waste. Without them, human life would not be possible. Countless good bacteria also live on and in our bodies. In the intestine, for example, numerous bacteria help us digest our food.

Page 2

Bacteria are the simplest life forms on earth. They are unicellular organisms, which means that they consist of only one cell. Bacterial cells differ in size and structure from plant cells, which you have already seen in the last chapter. In contrast to plant cells, bacteria belong to the prokaryotes. Thus, the genetic material of the bacterial cell “floats” in the cell plasma without a sheath. In addition, bacteria are much smaller than plant cells. They are only 2-3 micrometers in size, which is 2-3 millionths of a meter.

Some bacteria also have ring-shaped genetic components floating in the cell plasma, the so-called plasmids. The plasmids contain very important information and enable the bacterium to develop resistance to antibiotics, which means that the bacterium becomes well protected from the antibiotic to the extent that the bacterium can no longer be harmed by the antibiotic. It can happen that the medicine prescribed by the doctor will no longer help the patient with tonsillitis because it cannot fight the disease-causing bacteria.

Page 3

The plasmid rings can even be exchanged between two bacteria and thus spread. This exchange is of course a problem when fighting diseases caused by bacteria. In the beginning, it may only be a certain bacterium against which the drug is no longer effective, but eventually an increasing number of different bacteria also become resistant to the drug.

The exchange of the plasmids takes place via so-called pili. The pili are small cell appendages with which bacteria can dock to other bacteria to exchange plasmid rings.

All reactions that are important for the bacterial cell, such as nutrient degradation and energy production, take place in the cell plasma. There are some similarities but also differences between eukaryotic plant cells and prokaryotic bacteria. One common feature is that bacterial cells, like plant cells, also have ribosomes, which you already learned about in the last chapter.

Page 4

To protect themselves from external attacks, bacteria have a cell wall that also provides strength. The cell is additionally surrounded by a kind of mucous layer to prevent dehydration. Inside, the cell wall is coated with a cell membrane, which has a very important function. It determines which substances are allowed to leave the bacterial cell or enter and remain in the cell.

Although a bacterial cell does not have organelles such as the Golgi apparatus, mitochondria or chloroplasts, a few species - like plant cells - can carry out photosynthesis.

In addition, bacteria often have bacterial flagella, which help them to move. The flagella are thread-like structures on the surface of the cell. They rotate like a propeller, creating suction or pushing the bacteria forward.

In the picture you can see all parts of the bacteria cell again.

A.2 Physics

Text 1: The relationship between heat and mechanical work

Page 1

The relationship between heat and mechanical work

Have you ever thought about what "heat" is? In the 18th century, people were still convinced that heat was a weightless substance called Caloricum. At that time, for example, the expansion of a thermometer fluid was explained as follows: at higher outside temperatures, more of this substance, (i.e. Caloricum) enters the pores of the thermometer fluid and the fluid therefore expands.

In the middle of the 19th century, however, a number of scholars concluded that heat is not a substance but has something to do with energy. Perhaps you have already learned the term energy. According to Lord Kelvin, energy is the ability of a body to perform mechanical work. Thus, it can be briefly described as the working ability or effectiveness of the body. For example, energy is motion energy, which is also called kinetic energy or potential energy. Potential energy, for example, is positional energy, like the apple that hangs from the tree has gravitational potential energy that is released when it falls from the tree.

Page 2

In 1842, the German physician, Robert Meyer, summarized his findings as follows: “Falling mass (i.e. the potential energy), motion (i.e. the kinetic energy), heat, light and electricity are one and the same object in different manifestations.”

About the same time, the English brewer, James Prescott Joule, suspected the existence of an equivalence between mechanical motion and heat. To investigate this relationship, he conducted an experiment in 1843. Mechanical energy was generated in a quantity of water that was thermally insulated, that is, protected from the outside temperature. Then, the temperature increase of the water was measured. In the figure you can see the test procedure. In a container is the insulated water. In the water, fins are connected to a paddle wheel, which when turned agitate the water.

Page 3

The shaft of the paddle is then turned by descending weights. The weights thus use their positional energy to turn the shaft. The fins of the paddle wheel then rotate in the water and heat it up. The temperature change can then be precisely measured in the tank. Based on the design, Joule was able to establish a very precise relationship between the potential energy, that is, the potential energy of the weights and the temperature increase of the water.

Page 4

The Principle of Conservation of Energy

This result made it possible to transfer assumptions that had already been formulated about mechanical energy to thermal energy.

For mechanical processes, it had already been determined that the energy of a closed system remains constant. In 1848, the German physicist Helmholtz formulated the general principle of conservation of energy in his writing, "On the Preservation of Force," which is formulated today as:

In a closed system in which any (mechanical, thermal, electrical, chemical) processes take place, the available total energy is retained.

Therefore:

E = E1 + E2 + E3 + ... + En

E Total energy

E1, E2, ... Energies in their different forms

Page 5

This sentence suggests that energy in a closed system is never lost and never generated: It can only be converted.

A closed system

What is a closed system? A closed system is a system from which energy is neither supplied from outside, nor is energy withdrawn from inside the system, that is, the system is sealed off and isolated from the environment. Therefore, no interaction can occur between the system and the environment.

In this closed system, however, the existing energy can be transformed, that is, from one form into another. An example of this transformation are the processes in a power plant. The chemical energy contained in crude oil is first converted into thermal energy in a power plant. This heat energy, which is also called thermal energy, is then converted in the generator into motion energy of rapidly rotating components, which is then converted into electrical energy.

Text 2: The inner energy of a system

Page 1

The internal energy of a system

In the last text, you already learned about energy and a closed system. In this text, you will now learn about the internal energy of a system.

As you know, energy occurs in different forms and can be transformed from one form into another. The energy can also be transferred from one system to another. For example, one system can release heat while reducing its own thermal energy, and another system can absorb that heat and increase its thermal energy.

The internal energy of a system is stored energy. It is therefore composed of the different forms of energy that exist within a system.

Page 2

Determining the internal energy of a system is not easy, but fortunately in most cases, this value is not needed. To describe the processes taking place, the changes in the energies of the bodies and systems involved can be recorded. For example, when a piston moves through a glass as the gas expands and cools, the temperature of the gas (i.e. its thermal energy) changes. But all other forms of energy that are part of its internal energy remain unchanged and have nothing to do with the flask being moved. For this reason, knowing the other forms of energy or the internal energy of the system is not important.

But as you can see from the example with the piston, the internal energy of a system can change. When work is performed by a system, its internal energy decreases. In contrast, when work is performed on a system, its internal energy increases.

Page 3

Not only the performance of work can change the internal energy of a system. When heat is added to or removed from the system, its internal energy changes. For example, if a gas is heated, its internal energy increases. If the gas releases heat to another system, the internal energy of the system decreases.

As you have already learned, all forms of energy can be converted into each other. The principle of conservation of energy you learned in the last chapter applies. In the principle of conservation of energy, one assumes a closed system. Thus, it is important that the system is closed. For example, when the electricity generated in the power plant (remember how that happened?) is used to heat a pot of soup, not all the chemical energy that was originally in the oil will arrive as heat in the soup. Why does this loss occur? When one form of energy is transformed into another, energy will always be lost, for example, through friction.

Page 4

This loss of energy means that a little heat is always released into the environment during this conversion. Therefore, the system, which consists of the power plant and the pot of soup, is not closed.

The fundamental theorem of thermodynamics

When a system in which only thermal and mechanical forms of energy occur, a special form of the principle of the conservation of energy applies. This principle is also called the fundamental theorem of thermodynamics.

The fundamental theorem of thermodynamics refers to the change of the internal energy U. Since only thermal and mechanical forms of energy occur in the special system, only the performance of work or the removal or supply of heat can change the internal energy of this system.

Page 5

The first fundamental theorem of thermodynamics

The change of the internal energy U is equal to the sum of the mechanical work A performed by or on the system and the heat Q supplied to or removed from the system.

The following applies:

ΔU = A + Q

The "triangle" in front of the U is the Greek letter Delta, which denotes a change, that is, a change in internal energy U.

# Appendix B

Table B1.1. Intercorrelations in the Massed Learning Condition (Lower Triangular Matrix) and the Distributed Condition (Upper Triangular Matrix) of All Measured Variables in Biology in Experiment 1

|  | 1 | 2 | 3 | 4 | 5 | 6 | 7 | 8 | 9 | 10 | 11 |
| --- | --- | --- | --- | --- | --- | --- | --- | --- | --- | --- | --- |
| Variable |  |  |  |  |  |  |  |  |  |  |  |
| 1. Learning outcome (immediate) | — | .51 | .24 | .4 | .33 | -.10 | .30 | -.42 | .37 | -.02 | -.03 |
| 2. Learning outcome (delayed) | .56 | — | .49 | .49 | .41 | .18 | .54 | -.29 | .51 | .23 | .07 |
| 3. Perceived difficulty | .36 | .42 | — | .44 | .20 | .20 | .32 | -.28 | .21 | -.18 | -.24 |
| 4. Self-predicted success | .24 | .07 | .37 | — | .20 | .14 | .16 | -.29 | .26 | -.04 | .20 |
| 5. Perceived similarity | .00 | .17 | .25 | .39 | — | .34 | .27 | -.36 | -.02 | .09 | -.21 |
| 6. Perceived learning coherence | -.08 | -.18 | -.17 | .30 | -.01 | — | -.06 | -.14 | -.05 | .17 | .00 |
| 7. Prior knowledge | .56 | .43 | .45 | .34 | .14 | .17 | — | -.43 | .21 | .12 | -.03 |
| 8. Grades | -.32 | -.12 | -.13 | -.08 | -.15 | -.13 | -.37 | — | -.24 | -.07 | .21 |
| 9. Reading ability | -.14 | .22 | .26 | -.21 | -.11 | -.16 | .11 | -.01 | — | .63 | .42 |
| 10. Working memory capacity | .18 | .04 | .49 | .27 | .11 | .20 | .24 | -.36 | .15 | — | .57 |
| 11. Reading strategy knowledge | .07 | -.17 | .07 | .11 | -.33 | .21 | .14 | -.34 | .17 | .32 | — |

Table B1.2. Intercorrelations in the Massed Learning Condition (Lower Triangular Matrix) and the Distributed Condition (Upper Triangular Matrix) of All Measured Variables in Physics in Experiment 1

|  | 1 | 2 | 3 | 4 | 5 | 6 | 7 | 8 | 9 | 10 | 11 |
| --- | --- | --- | --- | --- | --- | --- | --- | --- | --- | --- | --- |
| Variable |  |  |  |  |  |  |  |  |  |  |  |
| 1. Learning outcome (immediate) | — | .23 | .02 | -.06 | .27 | .07 | -.11 | -.34 | .59 | .34 | .07 |
| 2. Learning outcome (delayed) | .68 | — | .04 | .11 | .19 | -.31 | .39 | -.19 | .28 | .23 | .22 |
| 3. Perceived difficulty | .12 | .17 | — | .45 | .28 | .37 | -.04 | -.01 | -.17 | -.39 | -.28 |
| 4. Self-predicted success | -.10 | .03 | .57 | — | .41 | .33 | -.08 | .35 | -.24 | -.19 | .02 |
| 5. Perceived similarity | -.28 | .07 | .17 | .42 | — | .43 | -.27 | -.01 | .07 | .01 | -.1 |
| 6. Perceived learning coherence | -.32 | -.06 | .51 | .37 | .24 | — | -.28 | .21 | -.2 | -.37 | -.03 |
| 7. Prior knowledge | .34 | .58 | -.03 | -.07 | .14 | .02 | — | -.17 | .17 | .1 | -.19 |
| 8. Grades | -.30 | -.24 | .03 | .09 | -.14 | .04 | -.56 | — | -.26 | -.22 | .07 |
| 9. Reading ability | .09 | .19 | .08 | -.27 | .12 | -.24 | -.12 | -.01 | — | .63 | .4 |
| 10. Working memory capacity | .14 | .02 | .28 | -.13 | .05 | .16 | .33 | -.17 | .17 | — | .56 |
| 11. Reading strategy knowledge | .20 | .09 | -.04 | -.27 | -.18 | .12 | .08 | -.22 | .17 | .32 | — |

# Appendix C

Instructions Used in Experiment 1 (English Translations, the Original Instructions Were Provided in German)

The main instructions with explanations of the tasks were provided in Session 1. The instructor read the instructions out loud, while the participants read the instruction on screen and solved some examples. In the experimental Session 2-4, the participants received short instructions by the computer program before the respective task, for example “Now you are asked to read a text. Do you remember how you can use the keys to read the text?” or “Now we are going to ask you some questions about the text you just read.”. No further instructions were given. Furthermore, the participants did not know about the schedule, thus, when they will get which task. The students were informed about the domains (physics, biology) by the teachers ahead of the start of the experiments.

In the following, we will present a translation of parts of the main instructions given in Session 1.

C1. General instructions.

“[…] Your participation is very important to us. We need your honest opinion and your best performance because we want to find out how students learn best. […]”

C2. Instruction of Questions

“In some tasks we will ask you questions. I will now show you an example of what this looks like. Please press the “R” key to get to the example.

In the middle of the screen, you can see the question “Who is Harry Potter’s best friend?”. Below the question, you see a white box to type in your answer. When you have finished typing your answer, click on the button marked “Continue” to move on to the next question. You cannot go back to the questions once you clicked “Continue”.

Try to answer this question. Type your answer in the box below the question. When you are satisfied by your answer, click on the button marked “Continue”.

[…]

Very good. Please press the key with the “E” on it to go to the next page. In the next type of question you will get the question “What is the name of Harry Potter’s best friend?” and four possible answers: “Lucius Malfoy”, “Ron Weasley”, “Fred Weasley” and “Samuel Jackson”. Only one of these four answers is correct! Click on the answer you believe to be correct. If you are not quite sure, click on the answer you think is most likely to be correct. Again, if you click on the “Continue” button, you will get to the next question, but you cannot go back to the question afterwards.”

C3. Instruction of Text Reading.

“In the next sessions, we ask you to read texts. But these text will be presented to you in a special way. How this looks exactly, I will show you with an example. Now press the key “E” to get to the text. As you can see, you can only read the headline of the text yet. If you want to read the next sentence, press the right pointing arrow on the keyboard.

Now press the arrow pointing to the right. This will lead you to the next sentence. Very good, now you can read this sentence. […] As you can see, you can’t read the headline now. But if you want to read the headline again, you can press the key with the arrow pointing to the left. Press now the key with the arrow pointing to the left. Do you see? You can now read the headline again. By pressing repeatedly the key with the arrow pointing to the right, you can read through the text till the last sentence. If you would like to read a sentence again, you can use the key with the left pointed arrow to go back to this sentence.”
